# A Perspective on Late-Stage Aromatic C–H Bond Functionalization

**DOI:** 10.1021/jacs.1c10783

**Published:** 2022-01-27

**Authors:** Li Zhang, Tobias Ritter

**Affiliations:** Max-Planck-Institut für Kohlenforschung, Kaiser-Wilhelm-Platz 1, D-45470 Mülheim an der Ruhr, Germany

## Abstract

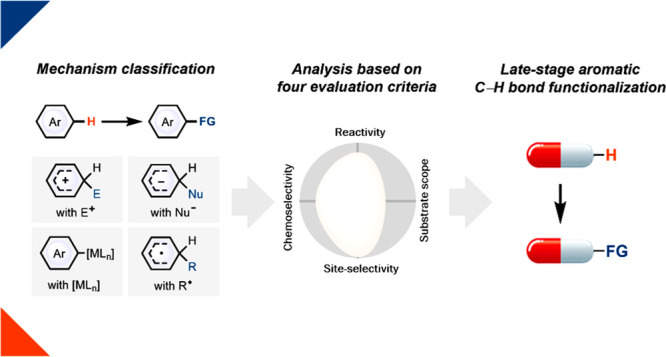

Late-stage functionalization of C–H bonds (C–H LSF) can provide a straightforward approach to the efficient synthesis of functionalized complex molecules. However, C–H LSF is challenging because the C–H bond must be functionalized in the presence of various other functional groups. In this Perspective, we evaluate aromatic C–H LSF on the basis of four criteria—reactivity, chemoselectivity, site-selectivity, and substrate scope—and provide our own views on current challenges as well as promising strategies and areas of growth going forward.

## Introduction

Late-stage functionalization (LSF) has been defined as “*a desired, chemical or biochemical, chemoselective transformation on a complex molecule to provide at least one analog in sufficient quantity and purity for a given purpose without needing the addition of a functional group that exclusively serves to enable said transformation*”.^1^ While LSF reactions do not have to be C–H functionalizations,^1^ every C–H functionalization on a complex molecule is a LSF and, thereby, potentially useful because sought-after molecules can be made quickly for use in diverse disciplines, such as drug discovery, materials research, and molecular imaging.^2−5^ Conversion of C–H bonds by late-stage functionalization (C–H LSF) takes advantage of the availability of complex molecules as starting materials—for example, when they are accessible from Nature, from a commercial supplier, or from a compound bank—so that new, previously inaccessible complex molecules can become accessible. The goal of LSF often is to quickly access derivatives that would be too challenging or time-consuming to make otherwise. The utility can be manifold, such as for structure–activity relationships of pharmaceutical candidates^2,3^ or to make a single new molecule with an unstable isotope that would not survive long enough for *de novo* synthesis.^4,5^ Once a desired compound is identified by LSF, it can frequently be made otherwise, more efficiently by *de novo* synthesis. While LSF cannot be substituted by *de novo* synthesis for all applications, in the majority of cases LSF makes it possible to access molecules that would not have been made otherwise. All complex organic molecules contain C–H bonds, so C–H LSF is a special case in the area of LSF, yet challenging because strong C–H bonds must be engaged selectively in the presence of a myriad of other functionalities. The field of C–H LSF is vast but can be separated into aliphatic and aromatic C–H LSF, of which we only discuss the C–H functionalization of arenes in this Perspective.

With the value of LSF by C–H functionalization established, and the ability to functionalize arenes by electrophilic aromatic substitution (S_E_Ar) reactions available for such a long time, why have conventional S_E_Ar reactions failed to be broadly useful for LSF? The concept of using C–H functionalization to generate diverse analogs of a complex molecule has been proposed for half a century,^6a^ but progress was slow initially. Some modern C–H functionalization^7−11^ reactions can operate under mild conditions and tolerate a variety of sensitive functional groups, which led to substantial advances of the field over the past two decades.^6b,6c^

Existing reviews on LSF have highlighted its utility and discussed various examples.^1^ In contrast to those reviews, we have elected here to analyze LSF in regard to challenges and future directions of different LSF reaction classes. We selected to analyze reactions on the basis of four criteria: reactivity, chemoselectivity, site-selectivity, and substrate scope. Although these criteria, in principle, are quantitative, the data for a global quantitative comparison does not exist. Therefore, in the interest of extracting guiding principles for the field, which is the purpose of this Perspective, we analyzed representative LSF reactions and attempted to provide a semi-quantitative evaluation that can provide meaningful characterization of the various reactions and put them in relation to each other, as well as to identify challenges and highlight advances for the field as a whole. In the Supporting Information to this Perspective, we have provided a semi-quantitative matrix that assigns for each of the four criteria one attribute, selected from *low*, *moderate*, *high*, and *excellent*, based on selection criteria provided in the Supporting Information. For example, *low* reactivity is assigned to reactions that require a reaction temperature higher than 120 °C and usually display less than 50% conversion or reaction times of longer than 3 days on many substrates, whereas *excellent* reactivity is assigned to reactions that proceed successfully to more than 95% conversion at 25 °C or below in less than 1 h and with catalyst loadings of 1 mol% or below; details are given in the Supporting Information. To rationalize our assignment, we have provided experimental detail and analysis for each of the 24 representative LSF reactions discussed in this Perspective. Because no uniform data exists that would allow for a universal, quantitative analysis, our analysis system is, by definition, subjective, and others may have assigned other values based on additional observables or other criteria. However, we would like to point out that the provided analysis is not intended as a comparative scoring board for past work but rather a useful tool to characterize the field as a whole and support a productive development going forward. For example, a “*low*” assignment in the criterion *substrate scope* might not necessarily mean an inferior characteristic compared to a “*high*” assignment but could simply rationalize a reaction that only proceeds on a well-defined but narrow substrate class, such as a reaction that proceeds on, for example, thiophenes but not on any other (het)arene. While limited in scope, such reactions can synthetically be extraordinarily useful. It is therefore not productive to compare reactions simply based on a single criterion. Definition of the criteria is as follows:

1. **Reactivity**: the intrinsic driving force combined with an appropriately small enthalpy of activation with which the C–H functionalization proceeds. The higher the conversion, the shorter the reaction time, and the lower the reaction temperature, the higher the reactivity of a reaction. For catalyzed reactions, the higher the turnover number and turnover frequency, the higher the reactivity.
2. **Chemoselectivity**: the rate of reaction toward C–H functionalization compared to the rates of reactions with all other parts of the substrate, often used synonymously with functional group tolerance. Comprehensive rate data is rarely provided, which is why chemoselectivity is typically assigned by evaluation of the functional groups that are tolerated in a given LSF. The reactivity of a reagent or catalyst with arenes can often be increased, yet such an increase in reactivity often coincides with an undesired drop in chemoselectivity.
3. **Site-selectivity**: the ratio of the rates for formation of one constitutional isomer compared to all others. In arenes, the distinction between *ortho*, *meta*, and *para* isomers is common but can become more complex for heterocycles, fused (het)arenes, and molecules with multiple (het)arenes. Although reactions that afford multiple constitutional isomers can be valuable to quickly generate molecular diversity,^12^ those reactions that afford only one constitutional isomer to the point that other isomers cannot be detected or be readily separated are often considered the most useful because well-defined, analytically pure compounds can be accessed reliably, as opposed to complex reaction mixtures that require laborious purification of various constitutional isomers.
4. **Substrate scope**: the degree to which a reaction can functionalize different types of arenes. High substrate scope, e.g., successful reaction with both electron-rich and -poor arenes as well as hetarenes, or arenes with different substitution patterns, can be desirable. Substrate scope and functional group tolerance (chemoselectivity) are not synonymous; chemoselective reactions, for example, can still have a small substrate scope.

Conventional S_E_Ar reactions typically do not score high in several, or in some cases even any, of the above-mentioned criteria, and even modern LSF chemistry cannot currently address all four criteria successfully at the same time. Broadly useful C–H LSF of arenes can therefore be considered as an unsolved problem, except for certain combinations of compound and reaction classes, with plenty of opportunities to make significant advances in the future.

A desirable simple though not straightforward solution to the field can be illustrated by a helpful albeit somewhat naive thought experiment: the development of a set of chemoselective, sufficiently reactive catalysts with large substrate scope that could each functionalize a single position selectively. For practitioners, three catalysts—one for *ortho*, one for *meta*, and one for *para* substitution of monosubstituted arenes—would be of high value due to the simplicity of the approach, and one might expect that such catalysts may also be successful for selective functionalization of di- or trisubstituted arenes and condensed arenes. Currently, we are far from reaching such a goal; no catalyst has been disclosed as of yet that would fulfill all four criteria for any position with a broad scope. What the field has accomplished so far, though, are several approaches that fulfill some criteria, often with concessions for the other(s), but those advances can be powerful and useful, even if currently only for a limited substrate scope.

With the four criteria as a measuring rod, we will discuss selected approaches from the past 15 years or so, arranged and classified by distinct reaction mechanisms, and explain how our own research has been guided or influenced by the analysis. This Perspective is our personal, biased, non-comprehensive view on how the field could move forward productively, what goals should be met by chemists who seek to make an impact in the field, and how selected past research has successfully addressed some of the challenges, while leaving other aspects still to be improved upon. While emphasizing reactivity, chemoselectivity, site-selectivity, and substrate scope as sole guiding principles, it has not escaped our analysis that other metrics, such as practicality, as determined, for example, by appropriate scalability, cost, and sustainability, are also inherently important to develop truly useful reactions. Although some practical reactions already exist, we will not include an evaluation based on current practicality given that, for the field as a whole, so many fundamental questions remain unanswered as of yet. Instead of burdening the current developments of the field with the need for the important aspect of practicality at this time, it may be instructive to first focus on the study of inherent reactivity principles to learn the fundamental lessons of the field.

Should the field develop a few general solutions that can address many problems, or should the goal be to develop a myriad of transformations, each individually ideally suited for a specific but rather narrow challenge? Generality is always desirable, but it is utopic to anticipate that the entire field will be served by just a handful of catalysts alone. Even if diverse substrate classes were suitably functionalized by a few, broadly useful catalysts, we have failed to discuss that the ensuing transformations are manifold for every single C–H functionalization; even if we can meet the goals for successful functionalization with one substituent to be introduced, there are numerous others. Many substituents merit incorporation, some of which provide sufficient material for entire research programs, such as fluorination, halogenation, oxygenation, amidation, amination, alkylation, perfluoroalkylation, and others. Different substrate classes with multiple potential reactive positions, potentiated by the different types of desired transformations, render the challenge quickly into a multidimensional matrix of problems that would require the same number of solutions (separate reactions that fulfill the four criteria). Such an approach seems impractical, at least difficult to navigate in the absence of artificial intelligence, even if all the reactions could be developed. In addition, the number of different catalysts and reagents that would need to be kept in stock to execute the reactions, if they could be identified, would be enormous.

Direct introduction of several useful substituents is certainly desirable, yet we also find the chemo- and site-selective introduction of linchpins by LSF particularly useful, as they dramatically reduce the number of problems that we need to find solutions to. A linchpin can be functionalized readily into a large variety of other substituents with, ideally, robust chemistry. In that sense, the LSF C–H functionalization step must only be carried out once, and the resulting molecule with an appropriate linchpin can subsequently serve as reliable starting material for a multitude of other transformations. As such, introduction of extremely versatile functional groups, or those that have the potential to be developed to be versatile, should, in our opinion, receive special attention. Transformations of the versatile groups are “just” functional group interconversions that have the potential to be more robust. Although the functional group interconversions no longer qualify as LSF, they can quickly be used to diversify, while only solving the challenge of LSF, the C–H functionalization step, once. Linchpins, among others, can be bromide, boryl substituents, or the thianthrenyl group, but also a transition metal. For example, a transition-metal-catalyzed functionalization of benzene affords a Ph–[M] organometallic that is synthetically versatile and more readily functionalized than benzene itself. There are not many robust linchpin substituents and even fewer reactions to introduce them that fulfill our four criteria. For example, there is currently no aromatic bromination reaction that meets the goals we have set for a truly useful C–H LSF; more specifically, the challenge of site-selectivity is not met by any bromination reaction that has a large substrate scope at the same time. Introduction of a linchpin followed by a subsequent functionalization reaction is less direct than the introduction of a substituent directly from the C–H group, and it depends on the application which approach is more desirable. Introduction of a variety of different substituents may benefit from a robust linchpin, while introduction of a single desired substituent on large scale would benefit from a direct C–H LSF.

To put a conceptual framework on the field of arene C–H LSF, we opted for a mechanism-based classification of different reaction classes. Several other sorting principles are conceivable, such as substrate class, reactive component to react with the arene, or product identity (which substituent is introduced in the LSF), all of which may be equally appropriate to present the field in an accessible format. In our analysis, the mechanism-based classification allows for a conceptual approach to identify and analyze some of the most relevant future opportunities that we aim to illustrate with a future-oriented Perspective, as opposed to a presentation of past accomplishments in a style attuned to a review or highlight. In the following, we will discuss distinct mechanism classes employed for C–H LSF, select examples to showcase the approach, and give our personal opinion on the different approaches with a focus on our own chemistry that we know best. We attempted to classify the approaches based on how well the four criteria—reactivity, chemoselectivity, site-selectivity, and substrate scope—are met, which can serve as a foundation for discussion, disagreement, and further refinement as the field progresses.

## Electrophilic Aromatic Substitution (S_E_Ar)

Electrophilic aromatic substitution (S_E_Ar) is one of the most widely utilized, practical, and cost-effective reactions to functionalize arenes.^13^ Functionalization proceeds via electrophilic attack of the arene π system to form a σ-complex, called Wheland intermediate, followed by deprotonation. Although the reactivity patterns have been widely explored by the community, reliable, predictable solutions suitable for LSF are rather scarce, owing to the often harsh reaction conditions that have conventionally been used to generate reactive electrophiles. Chemoselectivity can be an issue when the electrophile also displays undesired electrophilic or hydrogen atom transfer (HAT) reactivity, for example, for some halogenating reagents.^14^ Catalysts have been developed to counteract the chemoselectivity issues, but control of site-selectivity at the same time remains challenging. Site-selectivity can be achieved when the energies of the constitutionally different Wheland intermediates (e.g., *ortho* vs *meta* vs *para*) are sufficiently distinct, which, conventionally, can be achieved for *ortho* and *para* vs *meta*, or the reverse, but less so to distinguish *para* from *ortho* isomers. Formation of Wheland intermediates can be categorized into a conventional S_E_Ar mechanism via direct attack of an electrophile to the arene π system, a metal-based S_E_Ar based on electrophilic attack of a metal species, an S_E_Ar via charge-transfer (CT) complexes, an S_E_Ar via single electron transfer (SET) followed by radical recombination, and an S_E_Ar via enzymatic catalysis.

### Conventional S_E_Ar via Direct Attack

**Scheme 1:**
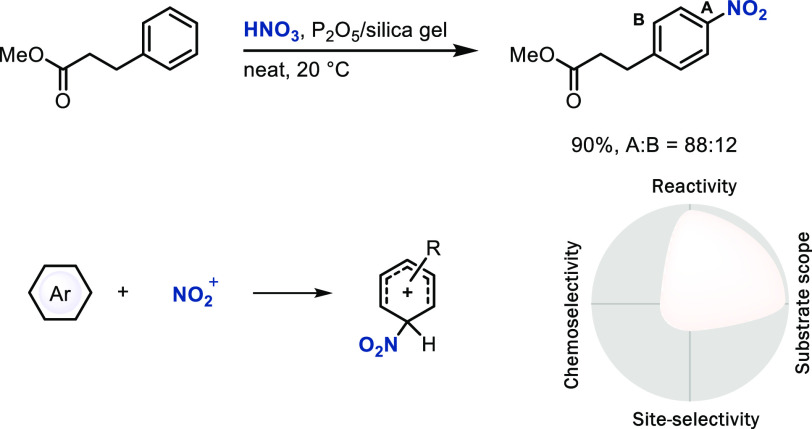
Electrophilic Aromatic Nitration

**Scheme 2:**
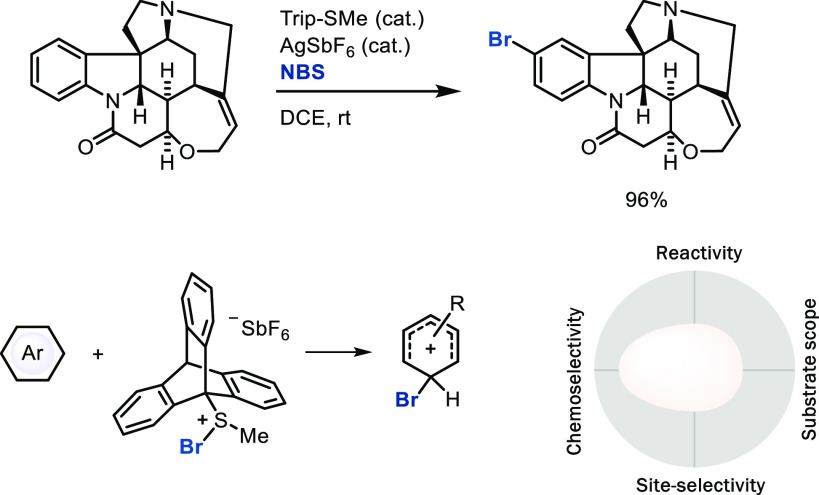
Late-Stage Electrophilic Aromatic Bromination with a Sulfide Catalyst

Conventional S_E_Ar often displays sufficient reactivity with broad substrate scope but low chemo- and site-selectivity. For example, nitration with nitric acid proceeds on both electron-rich and -deficient arenes but with few LSF examples (Scheme 1).^15^ Strong Brønsted or Lewis acids are used to generate reactive electrophiles, which has resulted in low chemoselectivity. Generation of reactive electrophiles by other means, for example, by the sulfide organocatalyst (Trip-SMe) for electrophilic bromination, through a reactive, charge-separated ion pair [Trip-S(Me)Br]^+^[SbF_6_]^−^, can increase chemoselectivity when compared to conventional reactions (Scheme 2),^16^ while site-selectivity still remains low in such cases. Site-selectivity is usually controlled by the relative stability of the Wheland intermediates, and the lack of differentiation based on substituent size in halogenation reactions generally results in low *para*/*ortho* selectivity on many substrates.^17^ Control of both chemo- and site-selectivity for a broad substrate scope is still a challenge worth studying.

### S_E_Ar via Electrophilic Metal Species

Electrophilic functionalization by reactive metal complexes has the advantage that there are ample opportunities to improve reactivity and site-selectivity by metal and ligand modifications, without change of the actual group to be transferred, which is possible only in a more limited sense for the modification of organic electrophiles. Moreover, the approach provides the option of catalysis and, based on the redox properties of the metal, atom-coupled electron transfer to make C–X bonds by external oxidants and X nucleophiles. It is important to point out the conceptual difference from other metal-catalyzed aromatic C–H functionalization reactions in the sense that at no point in the mechanism is a metal–aryl bond formed; instead, a high-valent metal–X complex functions as X^+^ electrophile, with concomitant reduction of the metal upon formal X^+^ transfer to the arene.

**Scheme 3:**
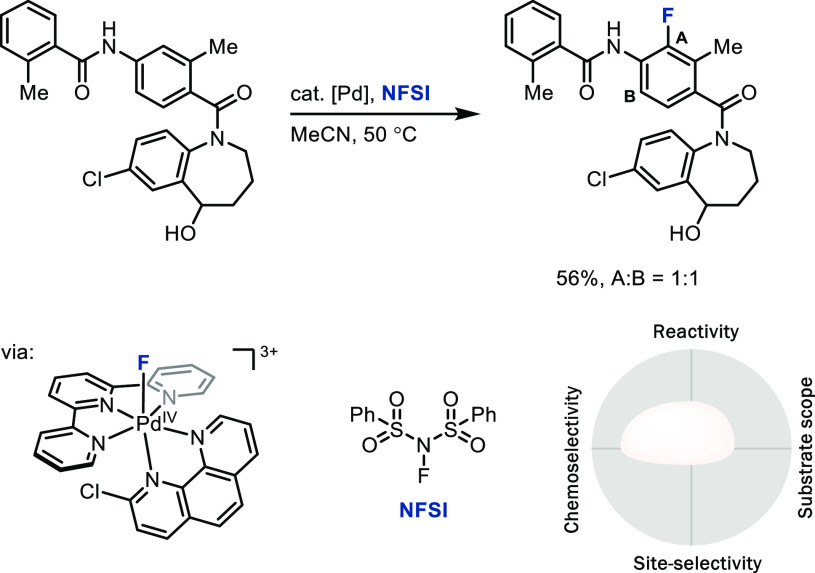
Late-Stage Electrophilic Aromatic Fluorination via a [Pd^IV^]–F Species

Our group approached electrophilic aromatic C–H fluorination with a palladium catalyst that can formally transfer an F^+^ to arenes through a mechanism that proceeds with lower barriers than have been achievable with conventional electrophilic N–F reagents directly (Scheme 3).^18^ The key intermediate in the electrophilic fluorination is an electrophilic Pd(IV)–F species, which may react with arenes in a fluoride-coupled electron-transfer mechanism. Potentially as a consequence of that mechanism, both chemoselectivity and substrate scope are larger than in direct fluorination reactions of arenes with commercial N–F reagents such as SelectFluor or NFSI, but the palladium catalyst appears incapable of inducing sufficient changes to the potential surface for site-selectivity. The approach, here specific for fluorination but also more generalizable, provides promising opportunities for future development: Both an increase in substrate scope and a similar reaction with fluoride in combination with an exogenous oxidant to arrive at structure like the Pd(IV)–F shown in Scheme 3 would be important steps forward in this field.

### S_E_Ar via Charge-Transfer (CT) Complexes

**Scheme 4:**
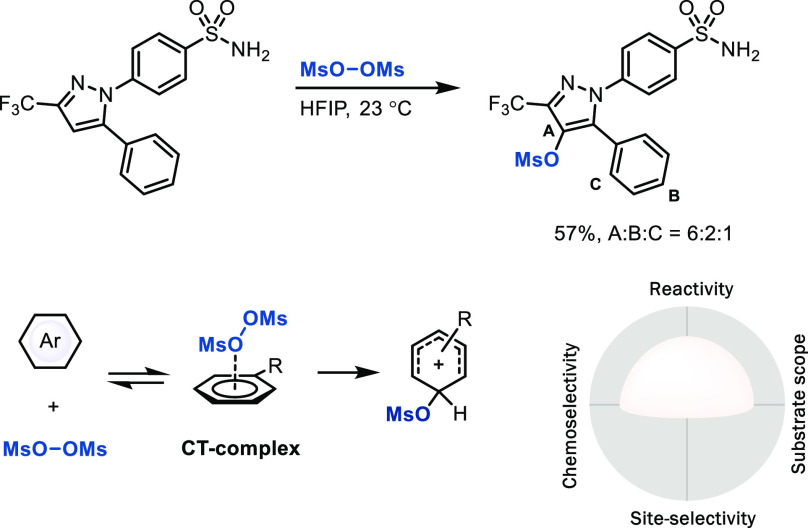
Late-Stage Electrophilic Aromatic Oxygenation with Bis(methanesulfonyl) Peroxide

Pre-association of reagents or catalysts with the π system of (het)arenes with subsequent pseudo-intramolecular (het)arene functionalization can lower selectively both enthalpy and entropy of activation for the C–H functionalization step in preference to other, deleterious side reactions and, thereby, is primarily promising to achieve an increase in chemoselectivity. We have attempted to make use of such an approach to better channel the otherwise rather indiscriminate reactivity of peroxides due to the formation of oxygen-based radicals and their high propensity of HAT reactivity in preference to desired arene functionalization. Oxygenation of arenes with peroxides was discovered more than half a century ago^19^ but usually suffers from low functional group tolerance and over-oxidation. Formation of CT complexes between (MsO)_2_ and the arene prior to C–O bond formation may lower the barrier for an electrophilic pathway as opposed to a radical addition, which distinguishes (MsO)_2_ from other peroxides (Scheme 4).^20^ The electron-withdrawing mesylate renders the products less reactive than the starting materials and can be used for further transformations, such as deoxyfluorination or phenol formation.^20^ Development of the CT concept has not yet been extensively explored for C–H LSF but is promising, especially if appropriate CT complexes with catalysis could be developed that are less dangerous and explosive than (MsO)_2_.

### S_E_Ar via Single Electron Transfer (SET)/Radical Recombination

**Scheme 5:**
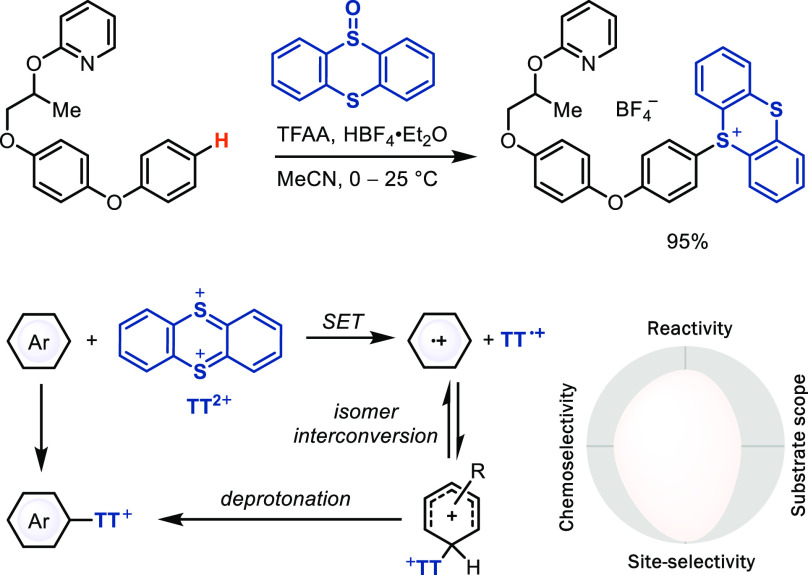
Late-Stage Aromatic C–H Bond Functionalization via Aryl Thianthrenium Salts

The development of modern S_E_Ar reactions has substantially increased chemoselectivity when compared to conventional reactions. However, most S_E_Ar LSFs that do not engage coordinating directing groups, including modern variants, still lack high site-selectivity. Our efforts in this field^12,21^ have resulted in the discovery of a novel thianthrenation reagent which can functionalize arenes with exquisite site-selectivity (Scheme 5).^22^ In most cases, both the *para*/*meta* and *para*/*ortho* selectivity are higher than 100:1, sometimes even higher than 500:1. The site-selectivity is possibly a consequence of a thianthrene (TT) dication intermediate that can react with arenes to Wheland intermediates, in which *para*/*meta* selectivity is determined by electronic factors and *para*/*ortho* selectivity is determined by steric factors.^23^ In addition, the reversible formation of the different constitutionally isomeric Wheland intermediates, which can dissociate by homolysis of the C–S bonds due to the stability of the persistent TT radical cation, allows the thermodynamic equilibration to the most stable Wheland intermediate before rate-limiting deprotonation (Scheme 5). Although radicals are present, thianthrenation was categorized as an electrophilic aromatic substitution because the initial C–X bond formation proceeds from two closed-shell species rather than a reaction between a radical and a closed-shell species. The resulting aryl thianthrenium salt is a linchpin for various transformations in cross coupling and photoredox chemistry and, due to its unusual electronic structure and properties, enabled several transformations that have not yet been achieved with other (pseudo)halides. For example, in addition to C–C,^22,24i,24j^ C–N,^24c^ C–O,^24d^ C–F,^24a,24e^ C–CF_3_,^24b^ C–B,^22,24f^ C–S,^22,24g^ C–Ge,^24h,24k^ and C–P^22^ bond-forming reactions, oxidative addition to Pd(0) of arylthianthreniums can occur in the presence of aryl bromides and triflates, and the cationic charge not only facilitates purification but also allows for generation of cationic Pd(II)–aryl complexes without strongly coordinating anions that enabled the first hydrogenolysis reaction of aryl (pseudo)halides with a homogeneous catalyst.^24l^ Thianthrenation is the most useful transformation our group contributed so far to the field of C–H LSF; however, we must point out that the subsequent transformations—substitution of the thianthrene linchpin—have generally low atom economy and generate TT as a byproduct that must be either recycled or discarded as waste.

### S_E_Ar via Enzymatic Catalysis

**Scheme 6:**
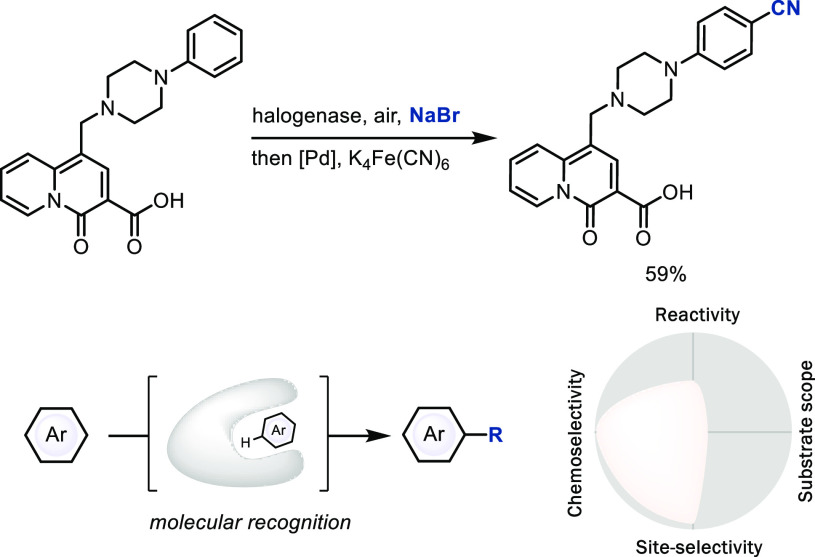
Enzyme-Catalyzed Late-Stage Functionalization of a C–H Bond

Biocatalysis with evolved enzymes can functionalize C–H bonds in high yields with high chemo- and site-selectivity that often go far beyond what is achievable with small-molecule catalysis or other small-molecule reagents.^11^ For example, the flavin adenine dinucleotide (FAD)-dependent halogenases (Fl-Hal) can selectively brominate a complex small-molecule arene in a C–H LSF (Scheme 6);^25^ a similar general bromination reaction without the use of enzymes is not yet known. However, while the substrate scope of enzymatic reactions is in principle vast, multiple rounds of time-consuming evolution may be necessary to optimize for a specific substrate or a specific site of functionalization.^26^ Yet, the opportunity to evolve a single mutant to achieve the same transformation but with high yet distinct site-selectivity and excellent chemoselectivity is still unmatched in small-molecule catalysis, albeit with rather limited substrate scope for every single mutant. Enzymatic C–H LSF can also proceed by nucleophilic aromatic substitution^27^ or radical aromatic substitution.^28^ Because the challenges and opportunities of enzymatic C–H LSF have commonalities when compared to small-molecule catalysis, we discuss them only here and do not repeat the analysis again in the following mechanism sections.

We are not expert enough to make any authoritative suggestions as to where the field of enzymatic C–H LSF should develop. From our point of view as small-molecule chemists, we would be excited about new enzymatic reactions that had large substrate scope and could readily furnish sufficient material for additional synthetic transformations, without the need for evolution by the user. We see a strong suit of enzymatic reactions particularly in those areas that are notoriously challenging to achieve with small-molecule catalysis, such as hydroxylation reactions.^29^ Phenols are often challenging to make with electrophilic reactions because the phenol products are more reactive than the arenes starting materials. While, electronically, the enzyme must deal with the same challenge, substrate specificity has been much more readily achievable in the enzymatic area.^26^

### Electrophilic Aromatic Substitution Analysis

Thianthrenation is special for C–H LSF via S_E_Ar reactions because it is highly site-selective and can generally be employed on more complex molecules than conventional S_E_Ar reactions because it is more chemoselective. Yet, thianthrenation shares the limitation of substrate scope with almost all other S_E_Ar reactions. Because the mechanism is, by definition, electrophilic in nature, reactions with electron-poor arenes, including the large and important group of six-membered *N*-heterocycles such as pyridine, and hetarenes derived from them, can currently not be achieved. A solution to this challenge is not obvious. Based on the analysis by Brown and Stock,^17^ we have introduced a linear free energy relationship that correlates *para* vs *meta* site-selectivity of any S_E_Ar reaction to the Hammett ρ value of that transformation.^23^ In other words, every S_E_Ar reaction with high site-selectivity known to date has a negative ρ Hammett value that is large, which means that the reactions with electron-poor arenes are too slow to be productive, hence the limited substrate scope. Electrophilic reactions that have sufficient reactivity to also engage electron-poor arenes display a negative Hammett ρ value smaller in magnitude, which will result in lower site-selectivity. Development of an electrophilic linchpin introduction that exhibits sufficient reactivity to also engage electron-poor (het)arenes despite a large, negative Hammett ρ value with high chemoselectivity for arenes and acceptable waste balance would be a major breakthrough in the field. For example, a highly site-selective bromination LSF reaction would be of great value. Claims of site-selectivity observed on structures that provide high intrinsic site-selectivity, such as anisole, aniline, or related compounds, are not very informative; we suggest to evaluate site-selectivity of ethylbenzene to obtain a true sense of the site-selectivity of an arene functionalization reaction. At least at the current state of analysis, it seems unlikely that a single reaction will be able to successfully functionalize carbocycles and electron-poor heterocycles site-selectively. The future of site-selective LSF by S_E_Ar may therefore realistically be in carbocycle functionalization. In that sense, it is fortunate that most carbocycles in complex molecules are rather electron-rich. Nevertheless, we recommend targeting reactions that can engage carboarenes from electron-neutral (e.g., benzene) to more electron-rich arenes to guarantee sufficient substrate scope. In such a scenario, we would concede that more electron-poor arenes and hetarenes are currently out of scope for site-selective S_E_Ar reactions.

## Nucleophilic Aromatic Substitution (S_N_Ar)

Traditional nucleophilic aromatic substitution (S_N_Ar)^30^ proceeds via nucleophilic *ipso* attack of a nucleophile on an electron-deficient arene that bears a leaving group to form a σ-complex, called Meisenheimer intermediate, followed by loss of the leaving group. Because a hydride cannot function as a leaving group as-is, a workaround for hydride elimination is a two-electron oxidation followed by deprotonation. The oxidation equivalent can be coupled to the arene or the nucleophile, as shown in the following two examples.

### Vicarious Nucleophilic Substitution (VNS)

**Scheme 7:**
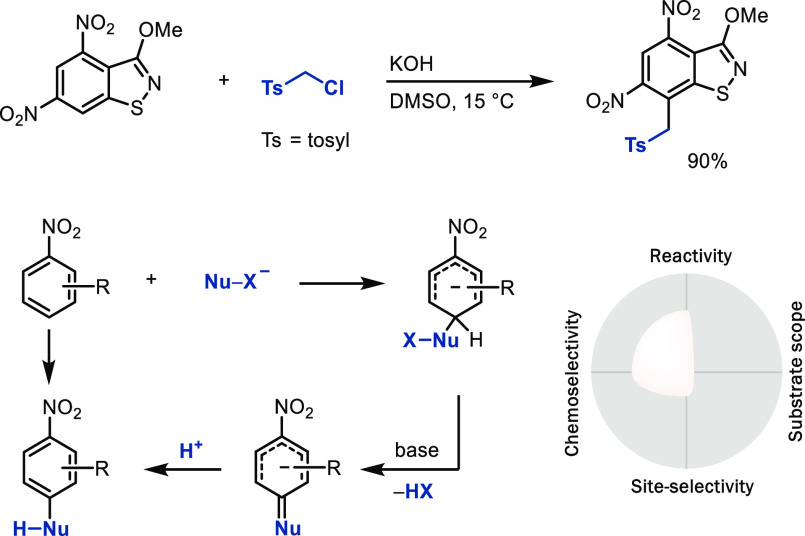
Vicarious Nucleophilic Substitution

In vicarious nucleophilic substitution, the nucleophile carries a leaving group X in the α position, so that HX can be eliminated upon nucleophilic attack to an electron-deficient arene, often an arene with a π-accepting functional group, such as nitro arenes (Scheme 7).^31^ Elimination initially forms an exocyclic double bond, and subsequent tautomerization restores aromaticity with a formal replacement of the hydride by a nucleophile. The two-electron oxidation equivalent is inherent to the nucleophile–X bond that is no longer present in the product. The requirement for the highly electron-deficient arene to ensure nucleophilic attack currently limits the scope of the reaction quite dramatically, and few LSF examples are published. Similar to VNS, in oxidative nucleophilic aromatic substitution, a σ-complex is formed by nucleophilic attack to an electron-deficient arene as well, which is then oxidized by an external oxidant.^32^

### Nucleophilic Substitution at Activated Pyridine

**Scheme 8:**
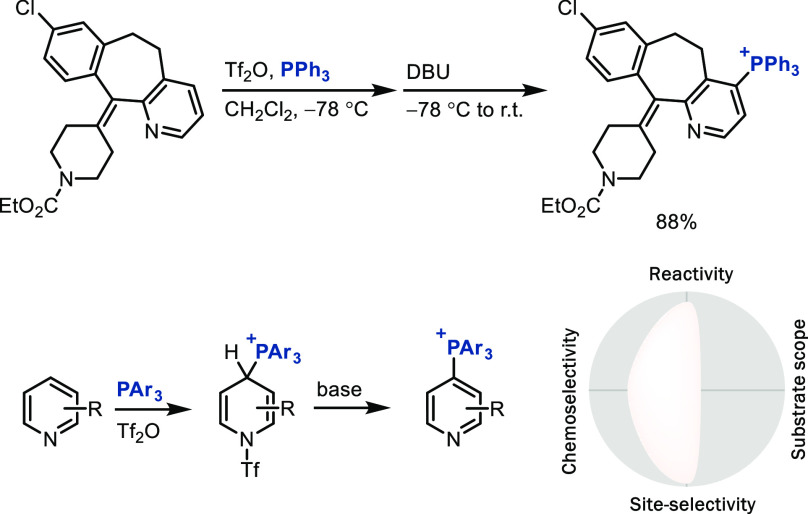
Late-Stage Aromatic C–H Bond Functionalization via Pyridyl Phosphonium Salts

Because six-membered nitrogen-based heterocycles are electron-poor, they are generally challenging to functionalize with electrophilic methods; thus, nucleophilic aromatic substitution appears as a promising alternative. However, direct attack of nucleophiles to pyridines is often limited to strong nucleophiles that may not be suitable for LSF. Moreover, products after addition are dihydropyridines, which require subsequent oxidation for rearomatization,^33^ so that the overall transformation requires multiple steps. With the same number of steps, the pyridine can also be activated prior to nucleophilic attack, which extends its reactivity and allows weaker nucleophiles to react with the π system of the pyridine. For example, the site-selective addition of triarylphosphines to pyridinium salts activated through triflation affords pyridyl phosphonium salts (Scheme 8); the phosphonium substituent can function as a linchpin for subsequent C–C, C–N, C–O, C–S, C–^2^H (or ^3^H), and C–CF_3_ bond formation.^34^ The oxidation equivalents in this transformation come from the pyridine activating reagents, in which a sulfonate sulfur(VI) is reduced as it leaves as sulfur(IV), a sulfinate leaving group. Although the substrate scope of this transformation is small, it targets a substrate class that is not yet accessible to many other regioselective C–H LSF and, as such, presents a useful complementary approach to electrophilic methods that best work on electron-rich arenes.

### Nucleophilic Aromatic Substitution Analysis

S_N_Ar reactions have been, both historically and in modern research, developed in smaller numbers when compared to S_E_Ar reactions, which may be explained by their much smaller substrate scope realized to date. Another reason may be the formal release of hydride from the Meisenheimer intermediate, which requires a coupled two-electron oxidation deprotonation that complicates development. However, it should not be underestimated that the potential for high-impact development in this field is high, not in the least because the substrate scope is often complementary to S_E_Ar reactions and, even if smaller than in S_E_Ar reactions, can often access structures that are out of scope for most if not all S_E_Ar reactions. Likewise, the site-selectivity differs from that of S_E_Ar reactions, which is easily explained by the mechanism, as opposing charges are stabilized at the same site as in S_E_Ar reactions. S_N_Ar reactions have therefore substantial potential and should not be neglected going forward. Because there are so few examples so far, every useful C–H LSF reaction in this area may be a major step forward.

## Arene Metalation

Under the topic of arene metalation we summarize all mechanisms that proceed through relevant organometallic intermediates, so at least one intermediate in the mechanism features a metal–aryl bond that is formed from a substrate aromatic C–H bond. Aromatic C–H functionalization reactions that proceed through initial arene metalation have probably contributed the largest number of new developments in the past couple of decades, also with progress to LSF. The advantage when compared to conventional S_E_Ar lies in the ability to deploy different (redox-active) (transition) metals with different sets of ligands that can elicit a wide array of different reactivity, e.g., substantially different mechanism pathways that can be accessed. It would also be possible to dissect the field into C–H functionalization reactions that make use of coordinating directing or templating groups and those reactions that do not; however, based on our mechanism-driven approach, we elected to sort the field by mechanism of C–H bond functionalization and categorized it into three metalation modes: electrophilic aromatic metalation, concerted metalation–deprotonation, and C–H bond oxidative addition.

### Electrophilic Aromatic Metalation

**Scheme 9:**
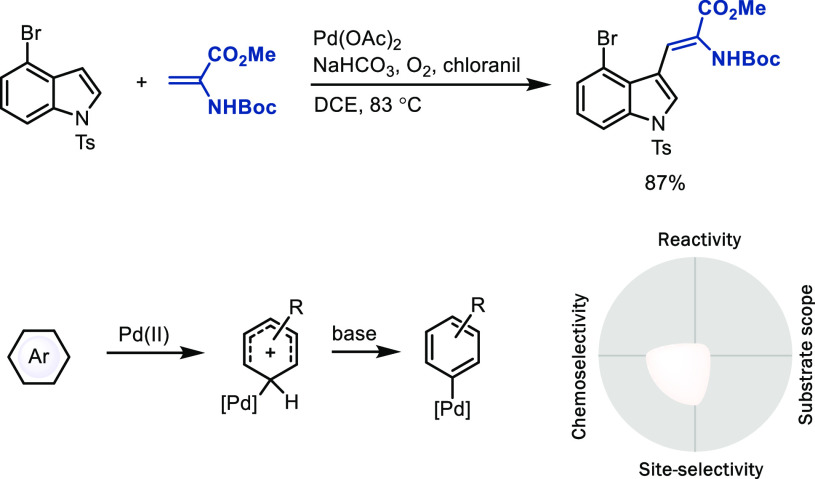
Functionalization of Indole Derivatives via Electrophilic Metalation

Early discoveries showed that electrophilic metal salts or complexes, such as those based on Pd(II),^35a,35b,35e^ Au(III),^35c^ or Hg(II),^35d^ can react with electron-rich arenes via an electrophilic aromatic metalation mechanism to generate Ar–[M] intermediates (Scheme 9). Electrophilic metalation proceeds analogously to S_E_Ar reactions, with the metal serving as the electrophile. Compared to more conventional aryl nucleophiles such as arylboronic acids or those obtained from decarboxylation of benzoic acids, direct C–H metalation circumvents the prefunctionalization of the arene yet also affords the Ar–[M] intermediates that may intercept other transition-metal-catalyzed processes such as cross-coupling and other C–X bond-forming reactions. However, the metals and ligand sets that are successful for C–H metalation are not always the same for diverse subsequent transformations.

### Concerted Metalation–Deprotonation (CMD)

Pure electrophilic metalation by metals is often limited to electron-rich arenes. However, some transition metal complexes, often with carboxylate ligands, are also able to functionalize less electron-rich arenes. For example, Pd(II) acetate, pivalate, and trifluoroacetate are catalysts that functionalize arene C–H bonds through synergistic electrophilic metal and internal basic ligand interactions.^36^ In other words, a basic carboxylate ligand on the metal serves as an internal base to deprotonate the nascent Wheland-like intermediate during an electrophilic metal interaction with the π system of the arene. This mechanism is therefore termed concerted metalation–deprotonation (CMD).^36d^ Site-selectivity in such cases is often more complex to predict than in pure electrophilic reactions because it is also governed by C–H acidity and steric hindrance.^35^ In many cases, it is not trivial to clearly distinguish electrophilic aromatic metalation from CMD or assign a reaction mechanism to either extreme, but the bottom line is that internal basic ligands such as carboxylates have substantially increased the utility of what was originally thought of as electrophilic metalation chemistry through assisted, intramolecular deprotonation.^36−40^

**Scheme 10:**
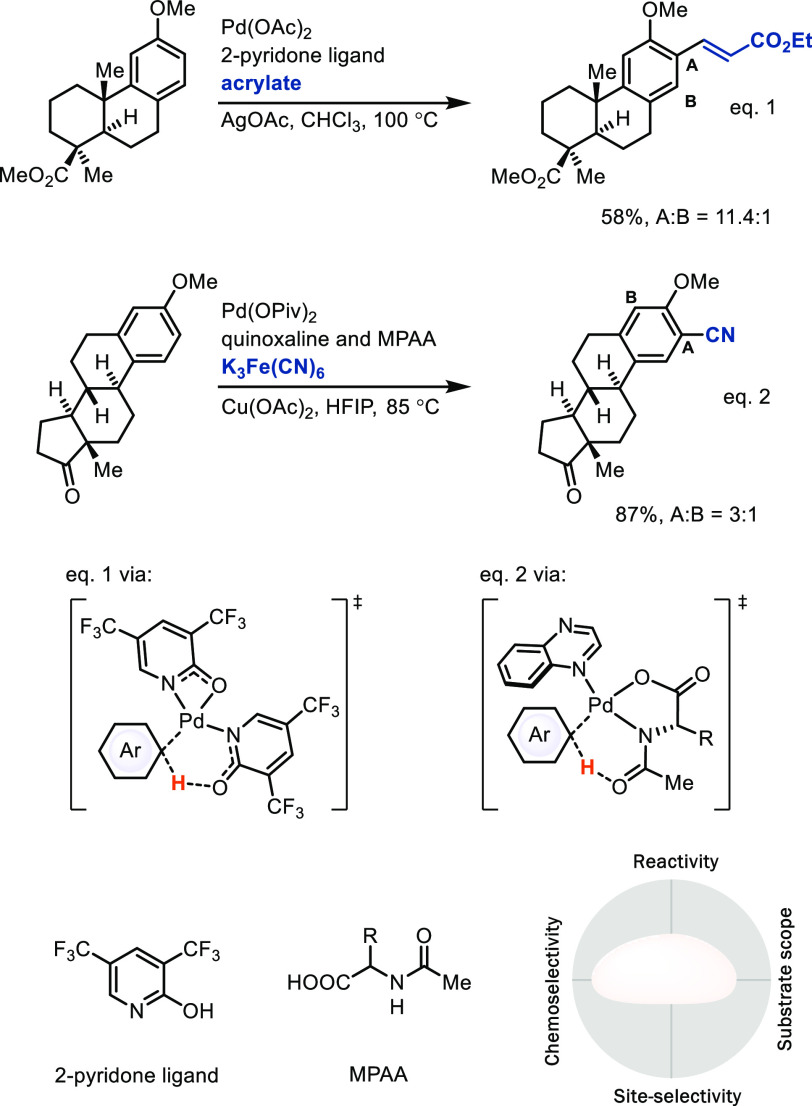
Palladium-Catalyzed Non-directed C–H Bond Functionalization Enabled by Ligand

*(A) Intermolecular arene metalation.* A major challenge in the field is the lack of reactivity of many catalysts with arenes, which requires high reaction temperatures or arene as solvent to obtain acceptable yields, both of which are unacceptable for LSF applications. A major advance in the field was the development of ancillary ligands that could accelerate the metalation step. Specifically, monoprotected amino acid (MPAA) and 2-pyridone ligands on palladium(II) accelerate C–H bond activation via CMD (Scheme 10, eq 1).^37^ Based on our own interest in the redox chemistry in similar reactions catalyzed by palladium carboxylates, specifically dinuclear Pd(III) chemistry,^38^ and the concepts developed by others in this area, most prominently by Sanford^39^ and Yu,^37^ we contributed a C–H cyanation (Scheme 10, eq 2).^40^ A remaining challenge in this chemistry deals with the modularity of the coupling partner, e.g., nucleophiles other than cyanide, not all of which can currently be added with the same efficiency. The *in situ*-generated Ar–[Pd] organometallic intermediate should, in principle, be useful as a broadly diversifiable linchpin, to be converted into a wide variety of substituents; however, the ligands and reaction conditions employed for efficient C–H metalation must also meet the requirements for efficient C–X bond formation, which is often not yet the case. For example, expansion of this chemistry to include catalysts for fluorination, amination, alkoxylation, and others would be a large step forward. Although site-selectivity can sometimes be controlled well through the CMD mechanism when large C–H acidity or steric hindrance differences are pronounced,^37,40^ generally, control of site-selectivity in this field is an unsolved problem for the most part.

**Scheme 11:**
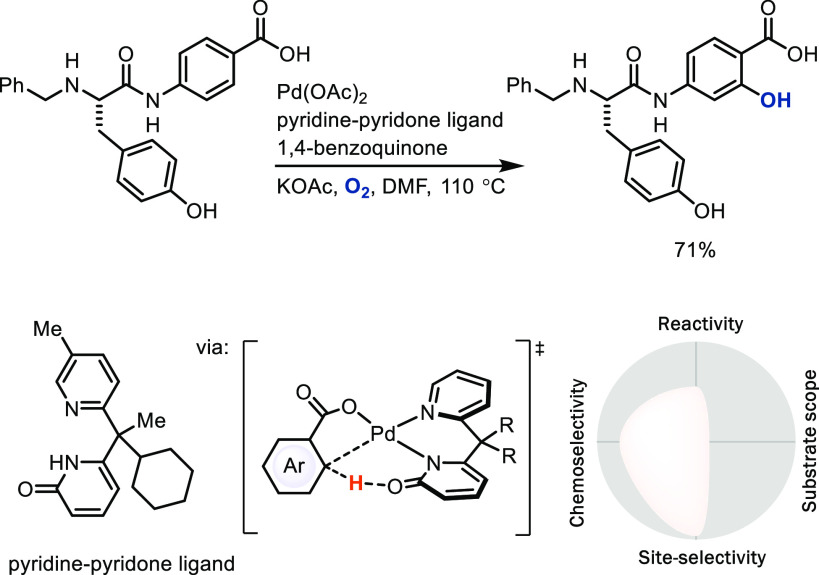
Palladium-Catalyzed *ortho* C–H Bond Hydroxylation of Aryl Carboxylic Acids

*(B) Metalation assisted by coordinating directing groups.* If coordinating directing groups or appropriate templates that direct the catalyst are present—covalently attached or transiently associated—the site-selectivity and reactivity of C–H bond metalation can be greatly increased compared to those of intermolecular metalation, with the caveat that such a coordinating group must be present in the molecule, which reduces the substrate scope to those compounds that contain such a group at the appropriate position. Development in the field has expanded the use of endogenous directing groups^41^ that are often found in relevant molecules such as carboxylic acids,^41a,41g^ amides,^41b^ hetarenes,^41d,41f^ or sulfonamides,^41c^ which can result in reliable and site-selective C–H LSF. Directing groups and template-assisted metalation direct the metal catalyst to a specific C–H bond or bonds; for example, a late-stage *ortho*-selective C–H bond oxygenation of complex benzoic acids is possible with a pyridine–pyridone ligand (Scheme 11).^41g^

**Scheme 12:**
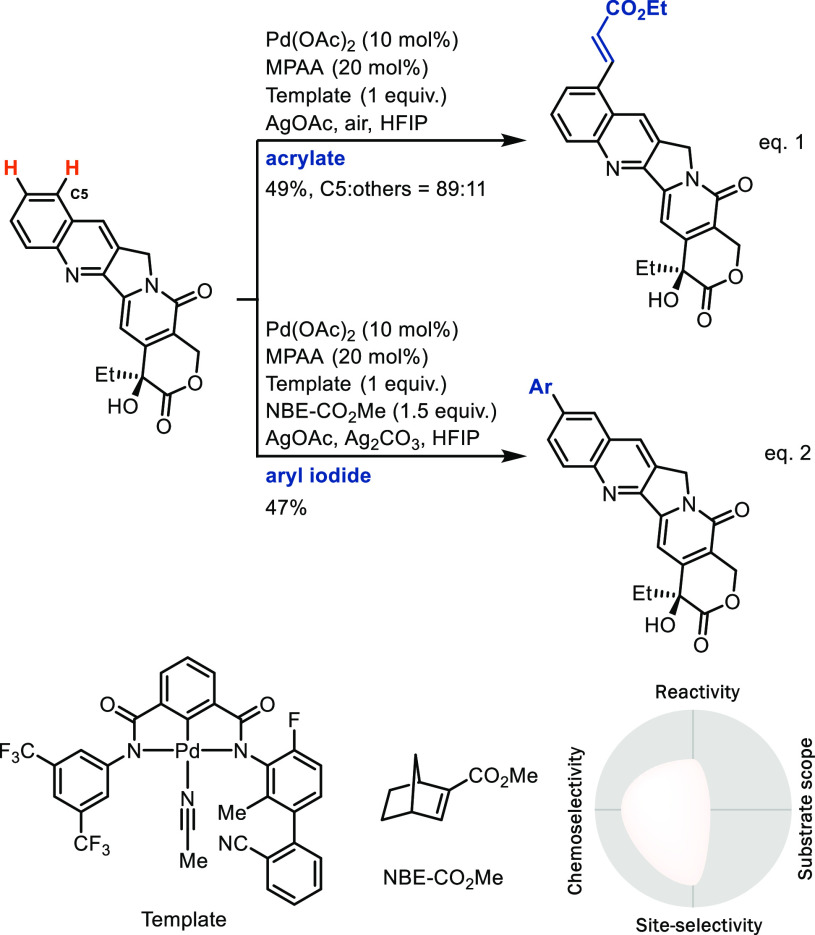
Palladium-Catalyzed Remote C–H Bond Functionalization of Quinoline via Template

Directed metalation–deprotonation has also been extended to *meta*-selective and *para*-selective reactions, albeit with more involved directing groups.^42c,42d^ The use of templates as non-covalent, transiently attached directing groups is an exciting development because the templates may eventually be used as catalysts and provide the opportunity to generate a family of templates to control site-selectivity for various different sites, without the need for a covalently attached directing group, yet still with the advantage of increased reactivity and high site-selectivity due to a pseudo-intermolecular C–H metalation event. An example of the use of a template is the divergent, site-selective remote functionalization of quinolines shown in Scheme 12.^42a^ In combination with a Catellani-based strategy^43^ through the use of norbornene (NBE), a palladium relay process enabled a site-selective arylation at the C6 position of quinoline (Scheme 12, eq 2).^42b^ We predict that further exploration of templates as catalysts for assisted metalation is a particularly promising strategy for site-selective C–H LSF.

### C–H Bond Oxidative Addition

**Scheme 13:**
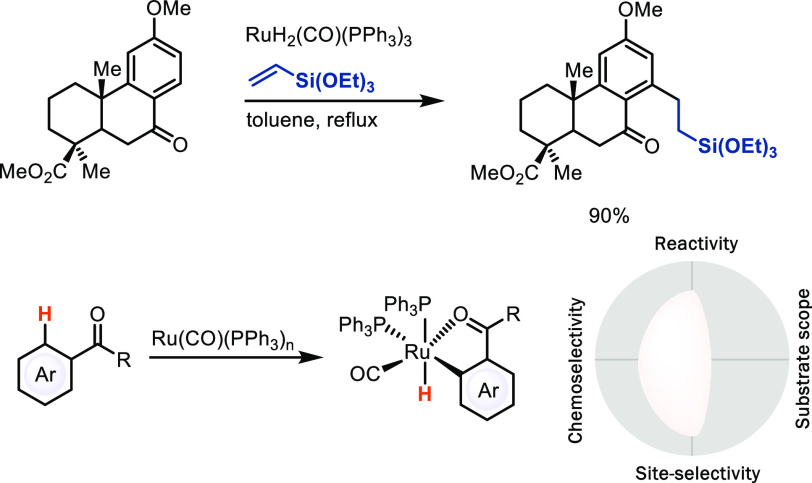
Ruthenium-Catalyzed Late-Stage Alkylation of C–H Bonds

Oxidative addition of C–H bonds is challenging yet offers large, complementary value to the other C–H functionalization methods due to its distinct potential. Metalation via direct oxidative addition can provide different site-selectivity and substrate scope from electrophilic aromatic metalation and CMD. A seminal example is the early work by Murai, who first disclosed a low-valent ruthenium catalyst for alkylation of aromatic ketones (Scheme 13).^44^ In contrast to the electrophilic and CMD mechanisms that require an electrophilic metal center, the metal complex reacts as a nucleophile in C–H oxidative addition, in which the C–H bond functions as the electrophilic reaction partner, with electron density in filled metal d orbitals that interact with the σ* C–H orbital. The resulting aryl–metal species can then undergo further transformations; for example, Ar–[Ru]–H proceeds with migratory insertion followed by reductive elimination to afford the alkylation product. The Murai reaction requires the ketone directing group for efficient, *ortho*-selective oxidative addition and high temperatures, which has limited its broad application in C–H LSF so far.

**Scheme 14:**
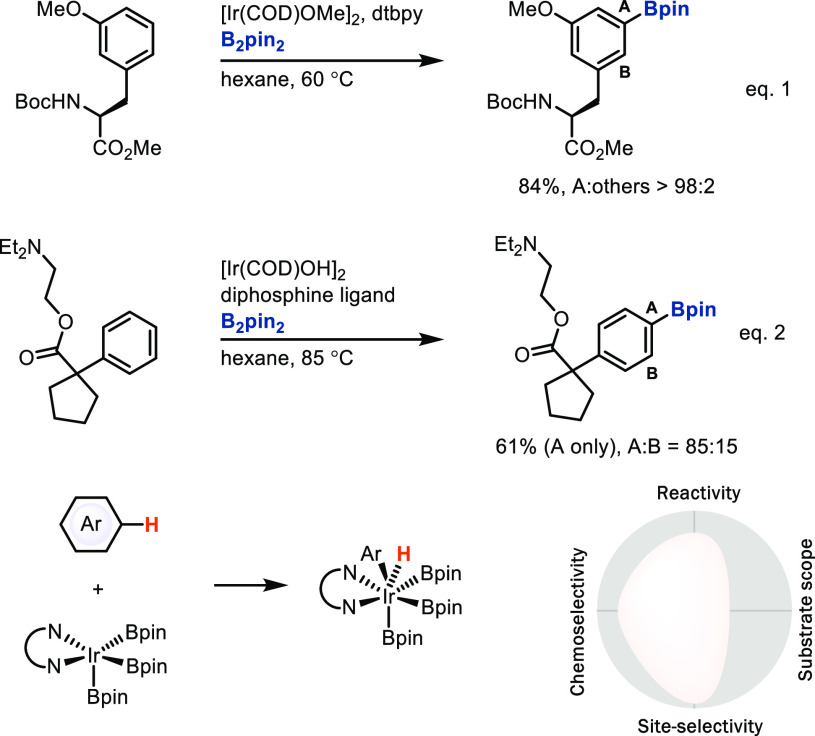
Iridium-Catalyzed Late-Stage Borylation of C–H Bonds

The iridium- and rhodium-catalyzed borylation^45^ and silylation^46^ reactions of arenes also proceed by C–H bond oxidative addition. In contrast to the electronically controlled selectivity for electrophilic metalation without coordinating directing groups, oxidative addition by iridium boryl complexes is sterically controlled and can give high, predictable site-selectivity for arenes that induce a high steric preference, such as 1,3-disubstituted arenes, to provide the 1,3,5-trisubstituted arylboronic acid derivatives (Scheme 14, eq 1).^45a−45d^ Although the substrate scope of Ir-catalyzed borylation is very broad, for many substrates that do not have the appropriate substitution pattern, site-selectivity is much lower (Scheme 14, eq 2).^45e^ In that sense, borylation is a special case in our diagrammatic analysis, for which we could also have selected to score with much larger substrate scope, however, with decreased site-selectivity. Given that we deem a highly site-selective reaction on a subset of appropriately substituted reagents more useful than a site-unselective reaction with large scope, we have opted to present the analysis as shown in Scheme 14. A substantial advantage of C–H borylation is the introduction of a versatile linchpin substituent—maybe the most useful linchpin at the current state of the art—which can readily and chemoselectively be functionalized into a myriad of other substituents.^47^

**Scheme 15:**
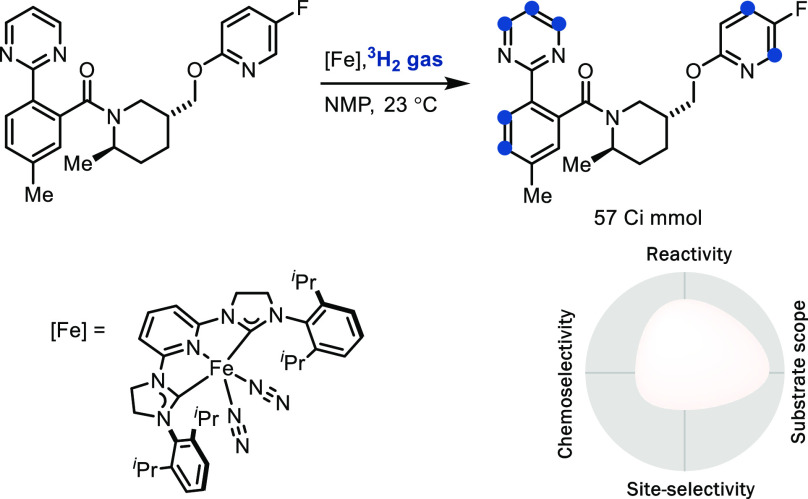
Iron-Catalyzed Late-Stage HIE Reaction

In addition to precious metals like Ru, Rh, and Ir, non-precious-metal catalysts can also activate C–H bonds via direct oxidative addition and sometimes afford different, complementary site-selectivity. For example, cobalt complexes can be used for C–H borylation of arenes, often with different site-selectivity, determined by relative acidity in addition to steric factors.^48^ Low-valent iron catalysts can also be useful for C–H bond functionalization and can accomplish a hydrogen isotope exchange (HIE) reaction^49^ (Scheme 15). The tritiation method can also be used for various classes of nitrogen heterocycles, yet the functional group tolerance can sometimes still be limited due to the high reactivity of the catalysts. In general, abundant-metal catalysis bears substantial potential, not only due to the obvious benefit of providing more sustainable solutions but also to afford different reactivity and selectivity than the precious-metal counterparts. A substantial hurdle to overcome for the future is to increase functional group tolerance for larger chemoselectivity.

### Arene Metalation Analysis

Sufficient reactivity, which can be increased by directing-group-assisted intramolecular C–H metalation, is still a challenge for aromatic C–H functionalization by electrophilic or CMD-based metalation in the absence of coordinating directing groups. In this context, the large field of Pd carboxylate-catalyzed C–H functionalization made a leap forward when it was found that MPAA ligands can significantly accelerate the metalation step to generate the [Pd]–Ar intermediate as linchpin for the introduction of various useful functional groups. Even so, it has been our experience that this subfield could become even more relevant if the reactivity of metalation could be further increased, while keeping appropriate chemoselectivity. Development of more reactive catalyst systems is certainly a worthwhile and promising goal, especially if a variety of transformations can be accomplished from the [Pd]–Ar intermediate.

Directed arene functionalization reactions are often highly chemo- and site-selective with appropriate reactivity. The major drawback is the small substrate scope, given that a directing group must be present. However, more and more intrinsic directing groups can be used in this chemistry that are already part of many complex molecules and, therefore, more useful. We also predict that further development in this area with weakly coordinating directing groups and non-covalent interactions such as hydrogen bonds, ion−π interactions, and Lewis acid–base interactions in C–H LSF will increase utility. Especially in combination with templates that can be used as catalysts, this branch of research is promising going forward because various complex molecules could be used for various site-selective C–H LSF, without the need to install additional directing groups stoichiometrically on the substrate. The use of different templates will be particularly useful, as reactivity can be tuned to various but single sites in the ideal case.

Expansion of the direct C–H bond oxidative addition mechanism to coupling reactions other than borylation and silylation reactions would be a dramatic advance in the field. Metalation by concerted C–H oxidative addition requires different properties of the catalyst than what is required for electrophilic activation or CMD. Because the metal must be electron-rich to accomplish the challenging C–H oxidative addition, it is conceptually difficult to accomplish oxidative transformations, such as halogenation or oxygenation, that employ electrophilic X^+^ reagents; the low-valent metal complexes generally required for C–H oxidative addition could be oxidized themselves by X^+^ before they engage in C–H oxidative addition. For that reason, borylation and, to a similar extent, also silylation or HIE reactions are somewhat special in this field. Not only can the reagent used for borylation, e.g., B_2_pin_2_, coexist with the catalyst in the oxidation state from which C–H oxidative addition occurs, but also the boryl ligands on the active catalyst are strong σ donors that increase the electron density of the transition metal, which increases the rate for oxidative addition.^45a−45c^ Moreover, it has been speculated that the empty p-orbital on boron in the Ir–trisboryl complex can participate in and facilitate C–H oxidative addition through contribution of a σ-bond metathesis mechanism.^45c^ These structural features are not readily accessible for other functionalizations, yet their successful exploitation, possibly through the design of appropriate ancillary ligands, would be a breakthrough in the field. Likewise, development of other borylation reactions that can complement the existing one with respect to the site-selectivity would further increase the utility. Even in the absence of such additional development, borylation is currently among the top methods for C–H LSF, as it can introduce, for a well-understood subset of (het)arenes, chemo- and site-selectively at a late stage, one of the most useful linchpin substituents in a reaction that is already practical when judged by the cost and reaction conditions.

## Radical Aromatic Substitution

Radical aromatic substitution has been recognized for a long time,^50^ and modern developments, especially in photoredox catalysis and also electrochemistry, have resulted in a renaissance of radical chemistry for (het)arene functionalization, also amenable for C–H LSF. Radicals frequently have orthogonal reactivity when compared to nucleophiles and electrophiles.^51^ Thus, high chemoselectivity can result because functional groups such as alcohols and amines that are often challenging for nucleophilic or electrophilic transformations are often well tolerated in radical mechanisms. Yet, polarity matching, e.g., the reaction of electrophilic radicals with electron-rich π systems and vice versa, is also important for radical reactions and often is the deciding factor for substrate scope and chemoselectivity.^52^ There are several ways to generate radicals, which we have categorized into conventional thermolysis, metal catalysis, electrochemical initiation, photoredox, and electrophotoredox approaches.

### Conventional Radical Substitution via Thermolysis

**Scheme 16:**
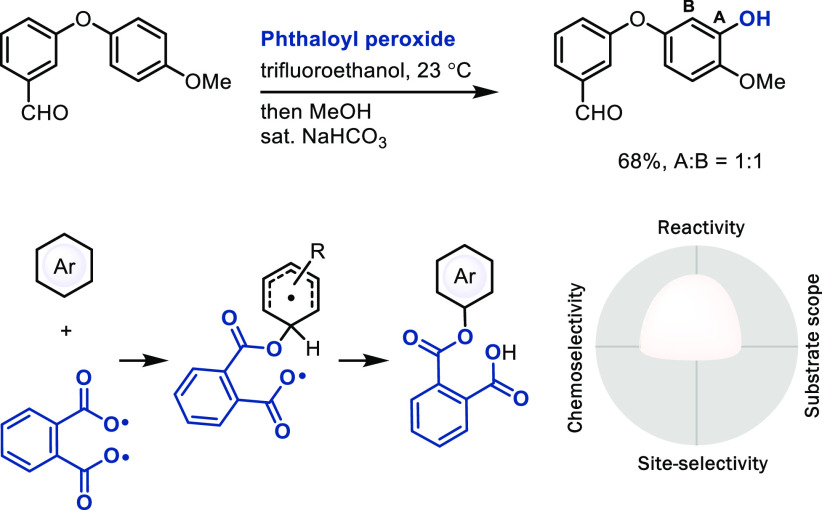
Late-Stage Oxygenation of Arenes with Phthaloyl Peroxide

Homolysis of peroxides is a common radical initiation reaction, while side reactions such as HAT to O-centered radicals can be a problem, and radical oxygenation reactions by peroxide often have low chemoselectivity. The phthaloyl peroxide reagent can produce an oxygen-centered diradical after homolytic cleavage (Scheme 16)^53^ that can add to arenes with fast, subsequent intramolecular H-atom abstraction from the resulting diradical. So far, relatively few radicals that are accessed by conventional thermolysis have been used for C–H LSF.

### Radical Substitution via Metal Catalysis

**Scheme 17:**
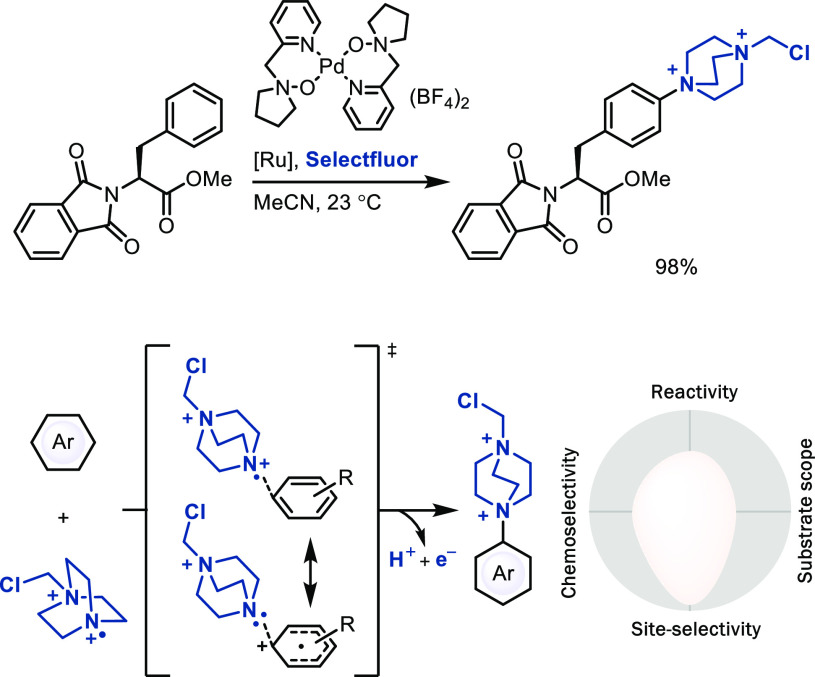
Late-Stage TEDAylation of Arenes

In most radical substitution reactions, different constitutional isomers can be observed. Our group developed a site-selective TEDAylation reaction (TEDA = *N*-(chloromethyl)triethylenediamine) that may proceed via a doubly cationic, radical TEDA^2+•^ species, generated through non-organometallic redox chemistry by a palladium catalyst from SelectFluor (Scheme 17).^21^ No arene–metal bond is formed, and the palladium(II) catalyst serves as SET complex to SelectFluor, which, upon fluoride elimination, generates the doubly cationic TEDA^2+•^ species for radical addition, followed by a second SET to generate a Wheland intermediate and regenerate the catalyst. Although the TEDAylation reaction does not produce a desirable functional group nor introduce a useful linchpin as far as we can tell now, we elected to cover it here as an unusual example of a highly positionally selective radical substitution mechanism. The selectivity was proposed to originate from a high degree of CT in the transition state of addition as a consequence of the high electron affinity of the doubly positively charged radical.^21^ However, we were not able to extend the CT concept to other radical additions while keeping the high site-selectivity of TEDAylation,^12^ which brings into question the generality of the concept. Better control and understanding of the underlying principles for synthetically useful site-selective radical arene substitution still remain unsolved challenges in the field.

### Radical Substitution via Electrochemical Initiation

**Scheme 18:**
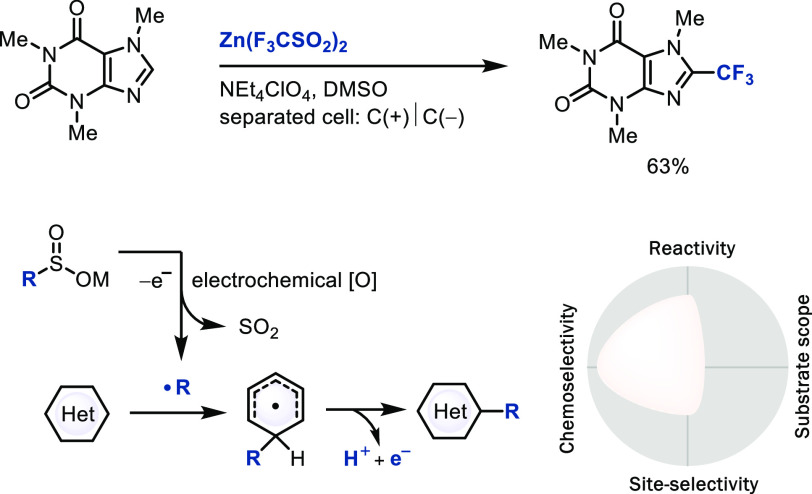
Late-Stage Electrochemical Trifluoromethylation of Hetarene with Zinc Sulfinate Reagents

Single-electron transfer to redox-active radical precursors by electrochemistry can generate radicals chemoselectively under mild conditions.^10^ Baran’s zinc sulfinate reagents, that can also generate radicals with peroxides as initiators,^54a,54b^ produce a broad range of alkyl radicals upon single-electron oxidation in an electrochemical setting for subsequent addition to heterocycles (Scheme 18). A wide range of heterocycles, including complex natural products, drugs, and other building blocks, can be functionalized chemoselectively without the use of protecting groups. The use of electrochemistry can, compared to the generation of radicals through peroxide initiation, further increase chemoselectivity, and thereby utility, for C–H LSF.^54c^ Single-electron oxidation of anions appears as particularly promising in this regard because the negative charge results in a high reduction potential for chemoselective oxidation.

### Radical Substitution via Photoredox Catalysis

**Scheme 19:**
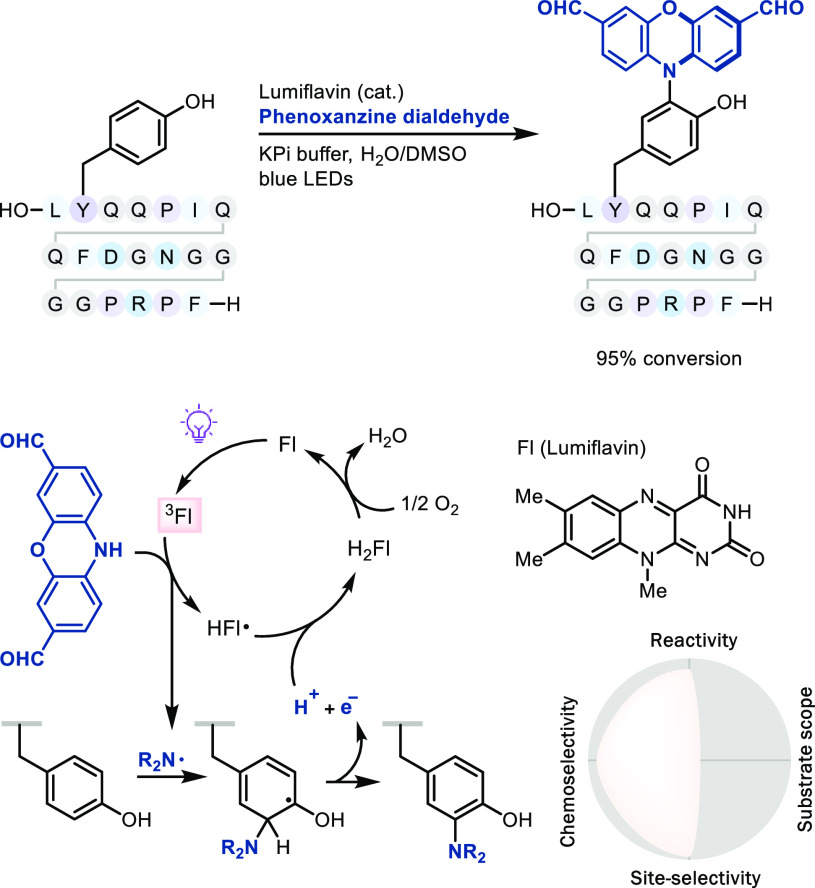
Late-Stage Amination of Tyrosine via Photoredox Catalysis

The development of photoredox chemistry has resulted in a series of new radical functionalizations for arenes and for the construction of C–C and C–heteroatom bonds.^9^ Selective chromophore irradiation by visible light, often with photoredox catalysts, allows for a mild, chemoselective transfer of substantial energy for subsequent productive chemistry,^55^ also in C–H LSF. For example, MacMillan developed a photoredox-based generation of the nitrogen-centered radical from phenoxazine dialdehyde for site-selective and chemoselective tyrosine modification^56^ in proteins (Scheme 19).^57^ The transformation is an example of how a particular chromophore can be excited in the presence of a variety of other groups. The large functional group tolerance of radicals toward polar groups, including the aqueous reaction medium, is apparent, yet polarity matching of radical and substrate can be made use of to achieve high chemoselectivity. The ability of light, also in combination with photoredox catalysts, to chemoselectively interact with well-defined chromophores, while most or all other parts of the reaction mixture, including large biomolecules, are transparent to the specific wavelength used, is of high value and bears large potential for future development. In this regard, introduction of small substituents, without the need for linkers, is an as of yet unachieved goal in this field that would be of substantial value, irrespective of the approach.

**Scheme 20:**
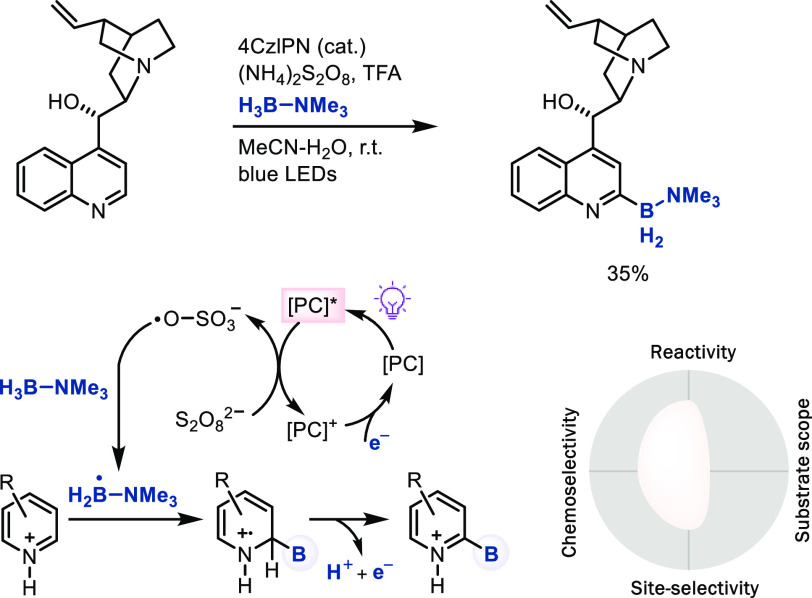
Late-Stage Borylation of Arenes with Amine–Borane

The addition of carbon-based radicals to protonated six-membered heterocycles such as pyridine was initially explored by Minisci and subsequently expanded by others.^58^ Protonation of the basic hetarene lowers the energy of the hetarene’s LUMO for better polarity matching with the electron-rich carbon radical. The resulting LUMO coefficients at C2 and C4 of substrates such as pyridine and quinoline are often similar, which often results in a mixtures of constitutional isomers. Instead of using carbon-based radicals, the Leonori group developed a pyridine borylation reaction using nucleophilic boron-centered radicals from an amine–borane complex, which also attacks the innately electrophilic position of the pyridine site-selectively (Scheme 20).^59^

**Scheme 21:**
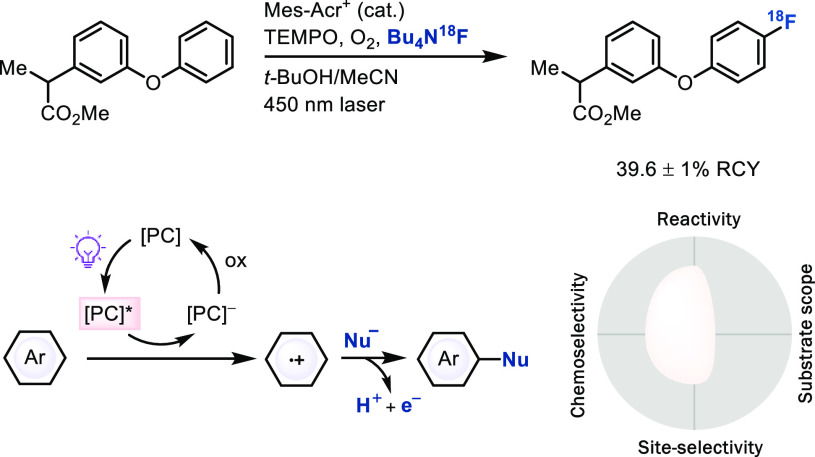
Late-Stage Radio-fluorination Reaction of Aromatic C–H Bonds via Photoredox Catalysis

As radicals can add to closed-shell π systems, closed-shell nucleophiles can add to arene radical cations, obtained by single-electron oxidation of typically electron-rich arenes with strong oxidants capable of oxidizing the neutral arene. The positive charge is best stabilized in the *ortho* and *para* positions of arenes with donor substituents, which explains the site-selectivity of the reaction. With appropriate photoredox catalysts that have sufficient oxidation potential, electron-rich arenes can be oxidized for subsequent, chemoselective nucleophile attack, as shown in the example of F-18 fluorinaion by Nicewicz (Scheme 21).^60^ Future development of stronger photo-oxidants could further increase the substrate scope of this reaction class, but retention of chemoselectivity under more oxidizing conditions will pose a challenge.^61^ Although currently with rather small substrate scope, the approach itself is highly promising because it allows for a different reaction mode that employs nucleophile addition with a coupled oxidant as opposed to more common and often expensive electrophilic reaction partners, which can be favorable, especially for those substituents that are more available as nucleophiles than electrophilic reagents, such as fluorine.

### Radical Substitution via Electrophotocatalysis

**Scheme 22:**
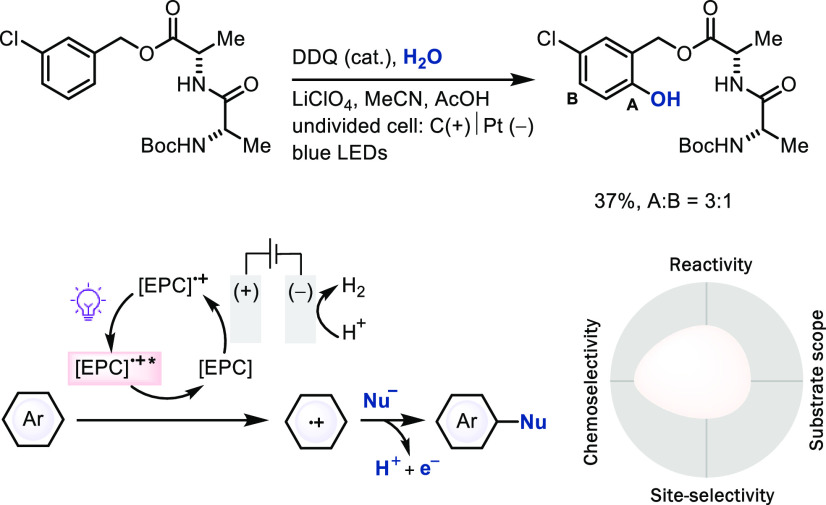
Late-Stage Hydroxylation of Aromatic C–H Bonds via Electrophotoredox Catalysis

The combination of electro- and photochemistry for LSF is relatively unexplored as of yet and allows for the generation of extreme potentials.^62c^ Electrochemistry can transiently generate intermediates, which then can be electronically excited to a highly reactive, non-equilibrium state with extreme redox properties. For example, arene radical cations can be obtained even from less electron-rich arenes. An electrophotocatalyst (EPC), such as the trisaminocyclopropenium ion (TAC)^62b^ and 2,3-dichloro-5,6-dicyano-1,4-benzoquinone (DDQ),^62a,62d^ can be accessed by anodic oxidation to generate strongly oxidizing photo-excited intermediates that can oxidize arenes to the corresponding arene radical cation intermediates, for subsequent nucleophilic attack (Scheme 22).^62d^ Proton reduction to H_2_ at the cathode often balances the redox equivalents for substrate oxidation, which can be problematic if high proton activity, e.g., low pH, is incompatible with the nucleophile. For example, the nucleophilicity of fluoride is dramatically decreased upon hydrogen bonding with H^+^, and carbanions as well as other nucleophiles with conjugate acids that are not sufficiently acidic cannot add when protonated. Likewise, the high redox potential generated by excited EPC may oxidize the nucleophiles such as amines, rather than the arene as a side reaction. Despite the challenges, the unusual properties that are achievable by electrophotoredox catalysis provide a promising foundation for future discovery in this relatively unexplored field and will likely result in new examples in the future.

### Radical Aromatic Substitution Analysis

Radical aromatic substitution distinguishes itself from the polar pathways discussed before in that the functional group tolerance, and hence chemoselectivity, is often different. Both nucleophilic and electrophilic groups such as alcohols, amines, and carbonyl compounds that can be problematic for polar mechanisms are often transparent to radical reactivity. Yet, polar effects must not be underestimated in radical transformations: polarity matches are important and influence rate constants of radical addition by many orders of magnitude.^52^ Such large difference in reactivity can be an asset, for example, when large biomolecules can be functionalized chemo- and site-selectively by radicals generated under conditions suitable to tolerate a myriad of other functionality, such as in a bioorthogonal photoredox event. Development of similarly selective reactions in this area will certainly add substantial value. Yet, development of chemistry, possibly by catalyst design that can further generalize radical reactivity, to extend the substrate scope could also increase the value of radical addition reactions. For example, extension of the addition of nucleophilic radicals to electron-deficient hetarenes such as pyridine as in some modern Minisci-type reactions to caboarenes would be a significant advance in the field, which may be difficult to address by optimizing polarity matching between radical and arene but potentially conceivable by catalysis. At the same time, development of radical substitution reactions that allow broadly useful yet selective linchpin introduction would be an additional large step forward.

## Outlook

We have provided our views on the different subfields, as well as a hopefully non-trivial analysis as to future goals and developments in the respective sections, and will not repeat them here. It is obvious that, at least in the near future, a small set of catalysts will not be able to offer solutions to the challenges outlined in this Perspective. However, it is not unconceivable that a subset of solutions, dozens maybe as opposed to hundreds, may successfully serve and cover a large space in the field. It is those types of transformations that will have the biggest impact. It is likely that no single reaction will cover the entire surface area of our analysis diagram, which we have provided for each selected transformation. It is our opinion that the one concession to make may be to accept smaller substrate scope. A large score in a single or two areas will not afford broadly useful reactions for C–H LSF, but if reactivity, chemoselectivity, and site-selectivity can be controlled to a high extent, even with limited substrate scope, such transformation will be broadly useful for C–H LSF. A variety of such reactions that are mutually complementary will offer a toolbox that can cover a wide range of substrates. We therefore recommend the development of reactions that can introduce linchpin substituents with a high driving force and high chemo- and high site-selectivity, even when they can only proceed on a limited but well-defined subset of molecules. The combination of such transformations will build a strong and reliable platform for C–H LSF.

## Supporting Information Available

Rationale and semiquantitative analysis for classification of each of the 24 representative LSF reactions with corresponding references (PDF)
