# Supplementary material for: A Perspective on Late-Stage Aromatic C–H Bond Functionalization
Source: J Am Chem Soc. 2022 Jan 27;144(6):2399–414. doi: 10.1021/jacs.1c10783 (PMC8855345; doi:10.1021/jacs.1c10783)
Supplement: Supplementary file 1 — ja1c10783_si_001.pdf [file ja1c10783_si_001.pdf]

SUPPORTING INFORMATION

A Perspective on Late-Stage Aromatic C–H Bond Functionalization

Li Zhang and Tobias Ritter*

Max-Planck-Institute für Kohlenforschung, Kaiser-Wilhelm Platz 1, D-45470 Mülheim an der Ruhr, Germany.

*E-mail: ritter@kofo.mpg.de

TABLE OF CONTENTS

TABLE OF CONTENTS 2

ANALYSIS BASED ON FOUR EVALUATION CRITERIA 3

General consideration for the analysis 3
Conventional electrophilic aromatic nitration 5
Electrophilic aromatic bromination with a sulfide catalyst 6
Electrophilic aromatic fluorination via a $[Pd^{IV}]$–F species 7
Electrophilic aromatic oxygenation with bis(methanesulfonyl) peroxide 8
Aromatic C–H bond functionalization via aryl thianthrenium salts 9
Enzyme-catalyzed C–H bond functionalization 10
Vicarious nucleophilic substitution 11
Aromatic C–H bond functionalization via pyridyl phosphonium salts 12
Functionalization of electron-rich (het)arenes via electrophilic metalation 13
Palladium-catalyzed non-directed C–H bond olefination enabled by ligand 14
Palladium-catalyzed non-directed C–H bond cyanation enabled by ligand 15
Palladium-catalyzed *ortho* C–H bond hydroxylation of aryl carboxylic acids 16
Palladium-catalyzed remote C–H bond olefination of quinoline via template 17
Palladium-catalyzed remote C–H bond arylation of quinoline via template and norbornene 18
Ruthenium-catalyzed alkylation of C–H bonds 19
Iridium-catalyzed borylation of C–H bonds 20
Iron-catalyzed hydrogen isotope exchange (HIE) reaction 21
Oxygenation of arenes with phthaloyl peroxide 22
Palladium-catalyzed TEDAylation of arenes 23
Electrochemical trifluoromethylation of hetarene with zinc sulfinate reagents 24
Amination of tyrosine via photoredox catalysis 25
Borylation of arenes with amine-borane 26
Radiofluorination reaction of aromatic C–H bonds via photoredox catalysis 27
Hydroxylation of aromatic C–H bonds via electrophotoredox catalysis 28

REFERENCES 29

ANALYSIS BASED ON FOUR EVALUATION CRITERIA

General consideration for the analysis

The evaluation of reactivity, chemoselectivity, site-selectivity, and substrate scope for the selected reactions would best be done by an objective, quantitative, and comparative analysis. Unfortunately, such a quantitative analysis is not possible at the current stage because the reactions that we have evaluated have not been conducted under identical conditions, or even with the same substrates. In addition, evaluation based on specific simple substrates for which data may be available for several reactions such as anisole, aniline or related compounds are not very informative when it comes to late-stage functionalization potential. Analysis of representative observables as discussed below served as a basis for a semi-quantitative scoring that can give a vague characterization of the reactions' attributes. It cannot serve as a quantitative comparison between reactions but provide an analysis of the remaining challenges of the field. This data, in our opinion, is more valuable than not providing any scoring system albeit subjective and prone to disagreement and error.

Evaluation criteria	Score	Description
**Reactivity**	*Low*	Reaction temperature higher than 120 °C or otherwise harsh reaction conditions are required for most substrates, and the reaction usually shows less than 50% conversion or longer than 3 days reaction time on many substrates.
	Moderate	Reaction temperature higher than 50 °C are required to achive more than 50% conversion for most substrates, or the reaction time is longer than 24 h. For a catalyzed reaction, the conversion is less than 50% at 1–5 mol% catalyst loading but synthetically useful yields can be obtained when the catalyst loading is increased to 5–20 mol%.
	High	More than 90% conversion can be achieved for the majority of substrates that participate successfully in the reaction. Reaction temperature is below 50 °C for most substrates. For a catalyzed reaction, the catalyst should have a turnover number of at least 20.
	Excellent	Most substrates that proceed successfully in the reaction are converted to more than 95% conversion at 25 °C or below in less than 1 h. For a catalyzed reaction, the loading of catalyst should be at 1 mol% or below.

**Chemoselectivity**	*Low*	The majority of functional groups other than the arene interfere with the reaction and cannot be tolerated. The reaction cannot tolerate other reactive C–H bonds, such as benzylic or allylic C–H bonds.
	Moderate	Most other C–H bonds are tolerated. The reaction tolerates a variety of functional groups but may not tolerate amines, alcohols, carboxylic acids, basic heterocycles, sulfides or other fuctional groups that have high reactivity with electrophilic, nucleophilic, or Lewis acidic and –basic reagents or catalysts.
	High	In addition to most functional groups, highly reactive functional groups such as amines, aldehydes, or carboxylic acids are tolerated to some extent, and reactions can be carried out without protecting groups on such functional groups. Additonally, several complex small molecules can be functionalized.
	Excellent	Various complex molecules with miscellaneous functional groups can be functionalized chemoselectively at the arene C–H bonds.
**Site-selectivity**	*Low*	Undesired constitutional isomers or functionalization at more than one site with a ratio of smaller than 4 : 1 (desired constitutional isomer : all other isomers) are observed for most substrates.
	Moderate	Undesired constitutional isomers or functionalization at more than one site with a ratio of higher than 4 : 1 but smaller than 10 : 1 (desired constitutional isomer : all other isomers) are observed for several substrates.
	High	Undesired constitutional isomers or functionalization at more than one site with a ratio of higher than 10 : 1 but smaller than 50 : 1 (desired constitutional isomer : all other isomers) are observed for most substrates.
	Excellent	Undesired constitutional isomers or functionalization at more than one site are not observed for most substrates. The desired product is produced in a ratio of larger than 50 : 1 (desired constitutional isomer : all other isomers).
**Substrate scope**	*Low*	The reaction can only functionalize a few specific types of substrates with a specific, required functional group, or substitution pattern, such as a transformation that can only functionalize nitrobenzenes, or a reaction that only works on 1,4-disubstituted benzenes.
	Moderate	One category of arene substrates can be functionalized, such as electron-rich arenes or electron-poor arenes.
	High	Several categories of arene substrates can be functionalized, such as electron-rich arenes, electron-neutral arenes, as well as some hetarenes.
	Excellent	Various categories of substrates including electron-rich, –neutral and –poor arenes as well as various hetarenes can be successfully converted. Many different substitution patterns are tolerated.

Conventional electrophilic aromatic nitration

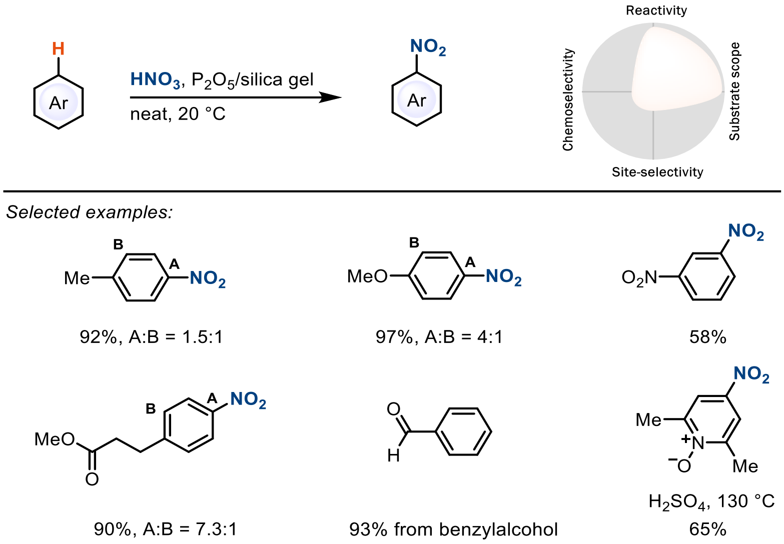

Evaluation criteria	Score	Analysis[1-3]
**Reactivity**	*Excellent*	1) Reaction temperature is 20 °C (or lower) for most substrates.[1]<br>2) Electron-rich arenes are converted to the corresponding nitroarenes in excellent yields within 2–7 min.[1]<br>3) For electron-poor arenes, even nitrobenzene, the reaction is complete within 20 min at 20 °C.[1]
**Chemoselectivity**	*Low*	1) When benzylalcohol was applied as substrate, alcohol oxidation is observed (benzaldehyde, 93% yield).[1]<br>2) Nitration of complex molecules under conventional conditions is rare.[1,2]
**Site-selectivity**	*Low*	1) The site-selectivity for toluene is *p*:*o* = 1.5:1.[1]<br>2) The site-selectivity for anisole is *p*:*o* = 4:1.[1]<br>3) Constitutional isomers are not observed in the nitration reaction of nitrobenzene.[1]
**Substrate scope**	*Excellent*	1) Electron-rich, –neutral or –poor arenes can be functionalized.[1]<br>2) Electron-poor hetarenes such as pyridines or pyrimidines are challenging substrates for electrophilic addition process, yet nitration can proceed successfully on such electron-poor hetarenes.[3]

Electrophilic aromatic bromination with a sulfide catalyst

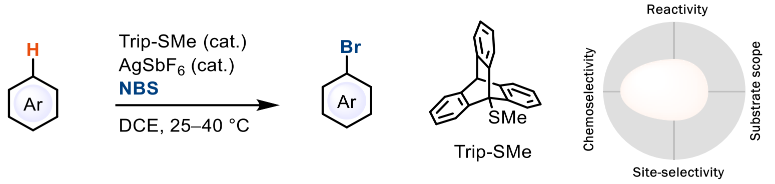

Selected examples:

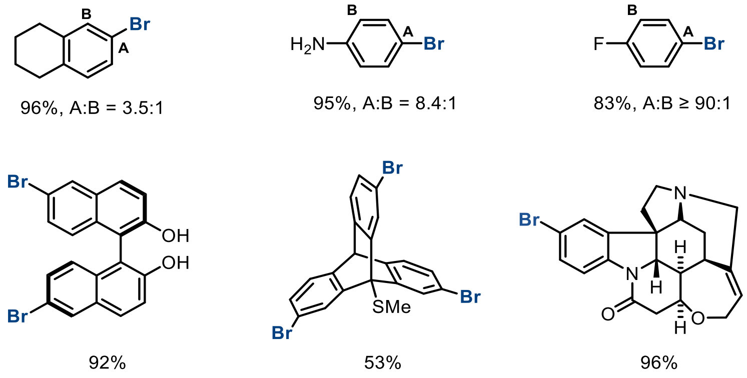

Evaluation criteria	Score	Analysis[4-6]
**Reactivity**	*Moderate*	1) Most reactions are complete within 24 hours at 25 °C.<br>2) The reaction requires 5 mol% Trip-SMe catalyst.<br>3) Bromination of the catalyst was observed, and the brominated catalyst can cause a significant drop in yield from 82% to 18% for *p*-xylene.[4,5]<br>4) Elevated temperature (40 °C) or longer time (24–46 h) are necessary for electron-neutral arenes.[4]
**Chemoselectivity**	*High*	1) Traditonal aromatic bromination catalyzed by $FeCl_3$ usually suffer from bromination at benzylic positions,[6] which was not observed in the sulfide-catalyzed bromination reaction.<br>2) Substrates with amines or phenols can be functionalized without protecting groups.[4]<br>3) The sulfide-catalyzed bromination can be applied to complex molecules containing a range of functional groups such as amides, esters, epoxides, amines, phenols, haologens, trifluoromethyls or hetarenes.[4,5]
**Site-selectivity**	*Moderate*	1) The site-selectivity for 1,2,3,4-tetrahydronaphthalene is C2:C1 = 3.5:1.[4]<br>2) The site-selectivity for aniline is *p*:*o* = 8.4:1.[4]<br>3) The site-selectivity for fluorobenzene is *p*:*o* ≥ 90:1.[4]
**Substrate scope**	*Moderate*	1) Both electron-rich and –neutral arenes can be functionalized.[4,5]<br>2) Electron-rich hetarenes such as indole, furan or thiophene can be functionalized.[4]<br>3) Electron-deficient arenes such as methylbenzoate are not reported as substrates for the electrophilic bromination reaction.[4,5]

Electrophilic aromatic fluorination via a [Pd^{IV}]–F species

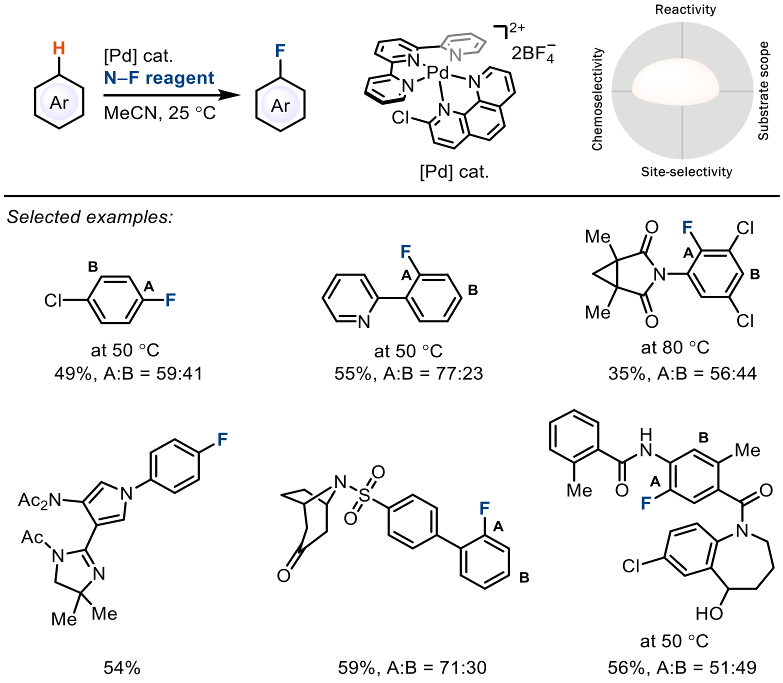

Evaluation criteria	Score	Analysis[7-9]
**Reactivity**	*Moderate*	1) For most substrates with an electron-donating group, the reactions are complete within 24 h at 25 °C.[7]<br>2) Elevated temperature (50–80 °C) or longer time (25–36 h) are necessary for electron-neutral arenes, chlorobenzene or complex molecules.[7]<br>3) The catalyst loading is 5–10 mol% for most substrates.[7]
**Chemoselectivity**	*High*	1) Substrates with alcohol groups can be functionalized without protection.[7]<br>2) Complex molecules containing ketones, esters, amides, halogens, sulfonamides, alcohols or hetarenes can be fluorinated.[7]
**Site-selectivity**	*Low*	1) The site-selectivity for chlorobenzene is *p*:*o* = 59:41.[7]<br>2) The site-selectivity for 2-phenylpyridine is *p*:*o* = 77:23 on the phenyl ring.[7]
**Substrate scope**	*Moderate*	1) Both electron-rich and –neutral arenes can be functionalized.[7]<br>2) Electron-deficient arenes such as methylbenzoate are not reported as substrates for the fluorination reaction.[7]<br>3) Hetarenes were not reported as substrates for the fluorination reaction.

Electrophilic aromatic oxygenation with bis(methanesulfonyl) peroxide

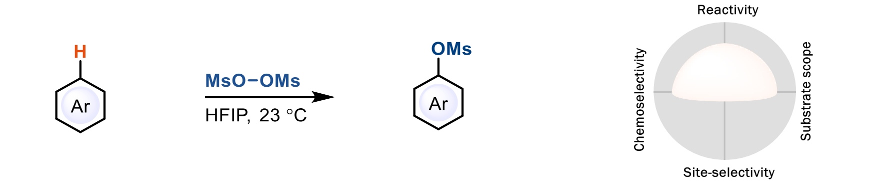

Selected examples:

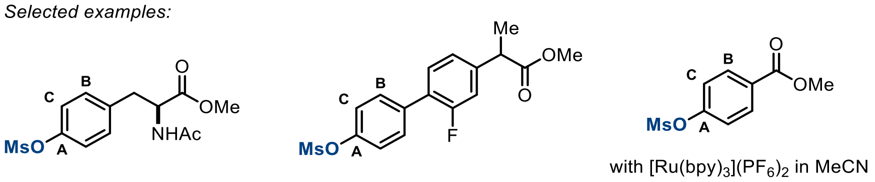

56%, A:B:C = 4:2:1 77%, A:B:C = 4:3:2 62%, A:B:C = 2:1:1

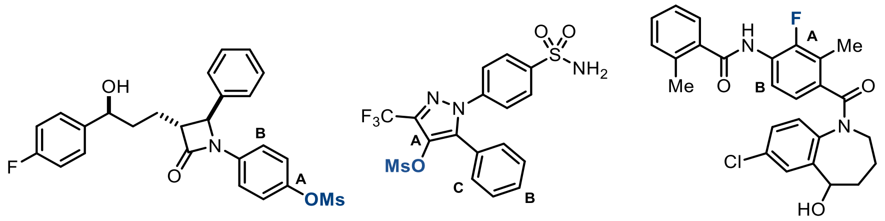

64%, A:B = 1:1 57%, A:B:C = 6:2:1 42%, A:B = 1:1

Evaluation criteria	Score	Analysis[10-12]
**Reactivity**	*High*	1) For most electron-rich and –neutral arenes, the reactions are complete within 12–24 h at 23 °C.[10]<br>2) In some cases, low temperature (0 °C) is necessary when adding the reagent $(MsO)_2$.[10]<br>3) Single-electron-transfer (SET) catalyst $[Ru(bpy)_3](PF_6)_2$ (2.5 mol%) is necessary for electron-deficient arenes.[10]<br>4) For most substrates, 1.1–1.3 equivalents of $(MsO)_2$ is required to achieve high conversion.
**Chemoselectivity**	*High*	1) Oxygenation of arenes usually suffer from overoxidation because the product phenol is easily oxidized,[11] which is not the case here.[12]<br>2) Substrates with alcohol groups can be functionalized without protection.[10]<br>3) Complex molecules containing amides, esters, sulfonamides, alcohols, trifluoromethyls, and hetarenes can be functionalized.[10]
**Site-selectivity**	*Low*	1) The site-selectivity for a protected amino acid, Ac-Phe-OMe, is *p*:*o*:*m* = 4:2:1.[10]<br>2) The site-selectivity for methylbenzoate is *p*:*o*:*m* = 2:1:1.[10]
**Substrate scope**	*High*	1) Both electron-rich and –neutral arenes can be functionalized.[10]<br>2) A single-electron-transfer (SET) catalyst is necessary for electron-poor arenes, yet oxygenation can proceed successfully on electron-poor substrates such as methylbenzoate.[10]<br>3) Hetarenes such as 2,5-dichloropyridines, azoles, quinoxalines, or thiophenes can be functionalized.

Aromatic C–H bond functionalization via aryl thianthrenium salts

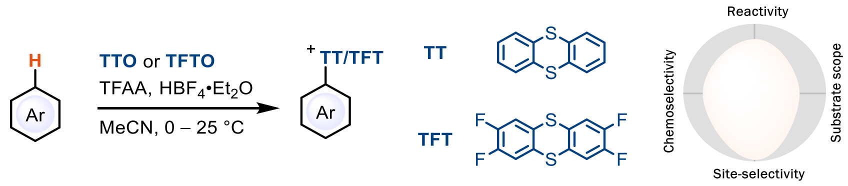

Selected examples:

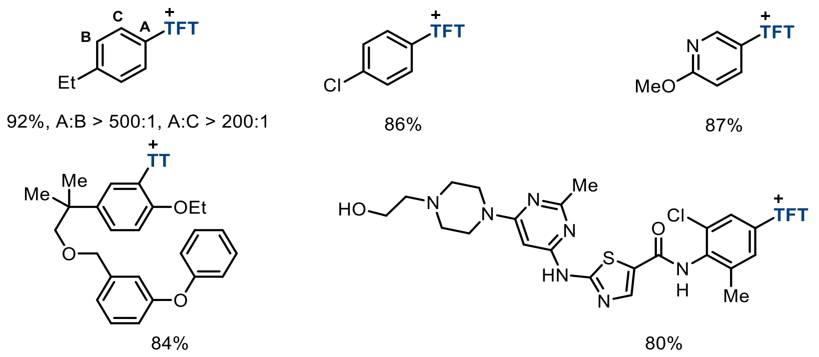

Evaluation criteria	Score	Analysis[13-15]
**Reactivity**	*High*	1) For most substrates, the reactions are complete within 1–24 h after warming from 0°C to 25 °C.[13]<br>2) For chlorobenzene, the thianthrenation reaction is complete within 3 h at 0–25 °C.[13]<br>3) High conversion of arenes can be achieved with 1.0 equivalents of thianthrene-*S*-oxide.[13]
**Chemoselectivity**	*High*	1) The key intermediates thianthrene dication[14] and thianthrene radical cation[15] are not reported to undergo hydrogen atom abstration, which is a common side reaction via electrophilic aromatic substitution mechanism.<br>2) Substrates with amines, phenols, alcohols or carboxylic acids can be functionalized without protecting groups.[13]<br>3) Complex molecules bearing amides, esters, sulfonamides, alcohols, trifluoromethyls, and hetarenes can be thianthrenated.[13]
**Site-selectivity**	*Excellent*	1) The site-selectivity for ethylbenzene is *p*:*o* > 500:1, *p*:m > 200:1.[13]<br>2) Constitutional isomers were not observed in the thianthrenation reaction of chlorobenzene.[13]<br>3) High site-selectivity is achieved in almost all cases, even for compounds containing several reactive positions.[13]
**Substrate scope**	*High*	1) Both electron-rich and –neutral arenes can be functionalized.[13]<br>2) Electron-deficient arenes such as chlorobenzene can be functionalized, but more electron-deficient substrates such as methylbenzoate are not reported as substrates for the thianthrenation reaction.[13]<br>3) Electron-rich hetarenes such as azoles, (benzo)furans, (benzo)thiophenes or 2-methoxypyridine can be thianthrenated.[13]

Enzyme-catalyzed C–H bond functionalization

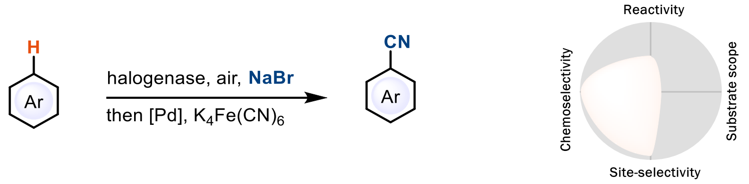

Selected examples:

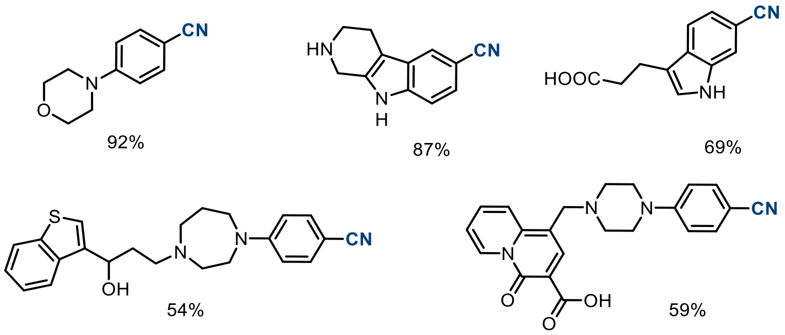

Evaluation criteria	Score	Analysis[16-18]
**Reactivity**	*Moderate*	1. Most reactions are complete within 12 h at 25 °C.[16]<br>2. For the natural substrates of halogenase, the conversion is high; but the conversion is much lower when the structure of substrates changes. For example, the conversion is 97±4% for tryptophan but 1.1±0.1% for anthanilic acid with wild-type SttH.[17]
**Chemoselectivity**	*Excellent*	1. Traditonal aromatic bromination reaction catalyzed by $FeCl_3$ usually suffer from bromination reaction at benzylic position,[6] which was not observed in the enzyme-catalyzed bromination reaction.[16]<br>2. Substrates with amines, carboxylic acids, alcohols, indoles, can be functionalized without protecting groups.[16]<br>3. Complex molecules bearing amides, amines, alcohols, and hetarenes can undergo the bromination reaction smoothly.[16]
**Site-selectivity**	*Excellent*	1. The site-selectivity is higly-dependent on the mutants used, the constitutional isomers are not observed when a proper mutant of the wild type enzyme was applied.[16]<br>2. Site-selectivity can be controlled by the variation of different halogenase, for example trytophan halogenation at C5, C6, or C7 can be achieved with enzyme PyrH, SttH or PrnA respectively.[16-18]
**Substrate scope**	*Low*	1. The natural substrates of the enzyme can be halogenated efficiently.[17,18]<br>2. Aniline, phenol, pyrole or indole derivatives can be halogenated with different mutants of flavin-dependent halogenases.[16,17]<br>3. Substituted phenols or polycyclic arenes can be halogenated with different mutants of heme-iron-dependent haloperoxidases.[18]<br>4. Electron-deficient arenes were not reported as substrates for the enzyme-catalyzed electrophilic bromination reaction.[16-18]

Vicarious nucleophilic substitution

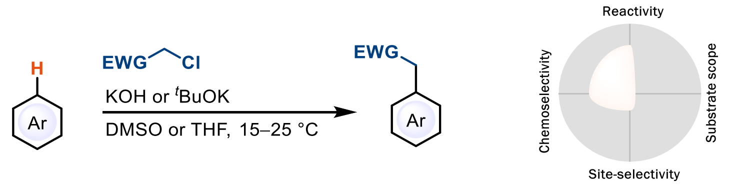

Selected examples:

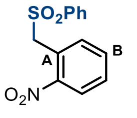

75%, A:B = 53:47

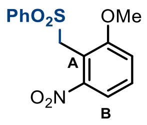

42%, A:B = 85:15

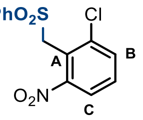

93%, A:B:C = 6:73:21

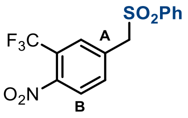

67%, A:B = 1:1

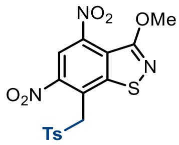

90%

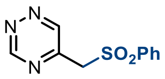

72%

Evaluation criteria	Score	Analysis[19-21]
**Reactivity**	*High*	1. Most reactions are complete within 2 h at 15–25 °C.[20-21]<br>2. Lower temperature (–30 °C) is also applicable to avoid undesired addition reactions at other electrophilic sites.[21]<br>3. High conversion can be achieved with 1.0 equivalents of nucleophiles.[19-21]
**Chemoselectivity**	*Moderate*	1. Addition of the carbanion to carbonyl groups will lead to Darzens condenzation product.[21]<br>2. Nucleophilic *ipso* attack to substituted nitrobenzenes via conventional S_NAr mechansms is a possible side reaction, but when a reactive leaving group was attached to the nucleophile, the side reaction can be avoided.[20]<br>3. Substrates with halogens, nitriles, trifluoromethyls or hetarenes can be functionalized.[21]
**Site-selectivity**	*Low*	1. The site-selectivity for nitrobenzene is *o*:*p* = 53:47.[21]<br>2. When the *para* position is blocked by substitutents, the VNS reaction is *ortho* selective.[21]
**Substrate scope**	*Low*	1. Nitrobenzene derivatives can undergo vicarious nucleophilic substitution reactions.[19-21]<br>2. Highly electrophilic heteroarenes which do not contain a nitro group such as quinoline-*N*-oxide, quinoxlaline-*N*-oxide, benzoxazole, benzothiazole, acridine or 1,2,4-triazine can also undergo the substitution reaction but others cannot.[21]

Aromatic C–H bond functionalization via pyridyl phosphonium salts

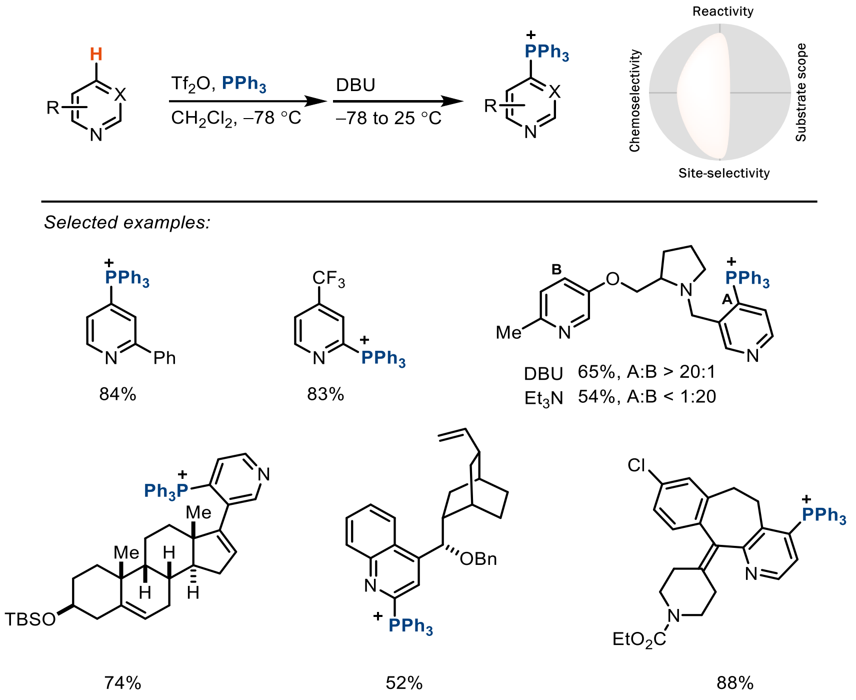

Evaluation criteria	Score	Analysis[22-24]
**Reactivity**	*Excellent*	1. Low temperature (–78 °C) is necessary when adding triflic anhydride, PPh_3, and base, the reaction is complete within 15–30 min after warming to 25 °C.[22,23]<br>2. High conversion can be achieved when only 1.0 equivalent of triflic anhydride and 1.1 equivalents of triarylphosphine are applied.[22,23]
**Chemoselectivity**	*Moderate*	1. Triflic anhydride must be applied to the substrate and may react with several functional groups such as aldehydes, ketones, alcohols *etc*.[22]<br>2. Several substrates with secondary or tertiary amines can be functionalized.[22]<br>3. Complex molecules with amides, esters, trifluoromethyls, halogens, alkynyls, alkenyls or hetarenes can be functionalized.[22]
**Site-selectivity**	*Excellent*	1. Only C4-substituted products can be observed when 2-, or 3-phenyl pyridines were applied as substrates. When the C4 of pyridines is substituted, the reaction is C2-selective.[22]<br>2. Constitutional isomers cannot be observed for most pyridine derivatives.[22]<br>3. When more than one pyridine rings exist in the molecules, site-selectivity can be controlled by base, phosphine, reagent order or acyl-blocking strategies.[24]
**Substrate scope**	*Low*	1. Pyridine derivatives can be functionalized.[22,23]<br>2. Substituted diazines, quinolines or pyrimidines can be functionalized.[22,23]<br>3. Functionalized arenes are not reported as substrates for the transformation.

Functionalization of electron-rich (het)arenes via electrophilic metalation

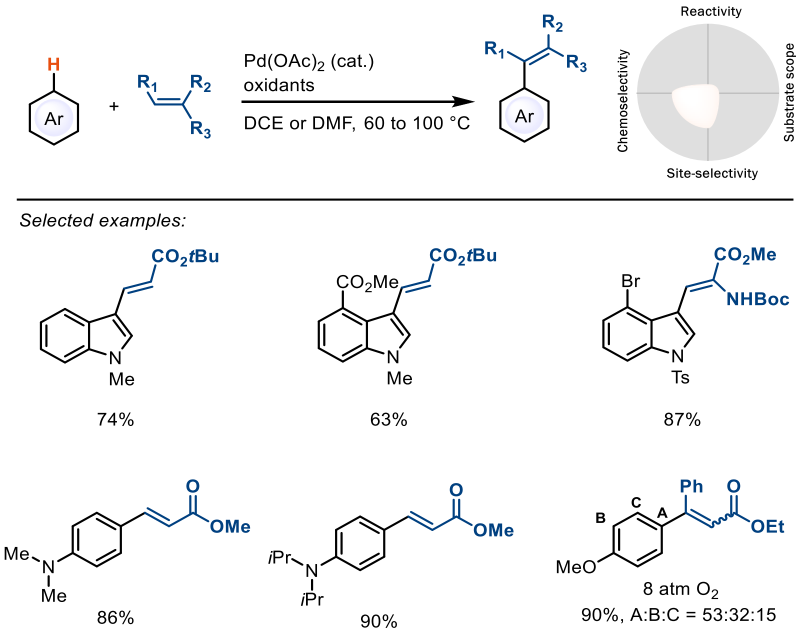

Evaluation criteria	Score	Analysis[25-28]
**Reactivity**	*Low*	1. For indole or aniline derivatives, most reactions are complete within 5–12 hours at 60–70 °C.[25,26]<br>2. For anisole, a large excess of arene (12–15 equiv.), high pressure of oxygen (8 atm), high temperature (100 °C), and long reaction time (25 h) are necessary.[27]<br>3. Stoichiometric amount of palladium acetate is required in the olefination of some functionalized indole substrates.[28]
**Chemoselectivity**	*Moderate*	1. Substrates with sulfonamides, amides, esters, ketones, halogens, alkenes, nitriles, amines or hetarenes can be functionalized.[25,26]<br>2. Substrates with unprotected amines, alcohols or carboxylic acids are not reported.<br>3. The electrophilic metalation process has been applied to the funtionalization of tryptophan derivatives and the total synthesis of Clavicipitic acid.[28]
**Site-selectivity**	*Moderate*	1. Constitutional isomers were not observed in the reactions of most indole or aniline derivatives.[25,26]<br>2. The site-selectivity for anisole is $p{:}o{:}m$ = 53:32:15.[27]
**Substrate scope**	*Low*	1. Indole or aniline derivatives can undergo the electrophilic metalation process followed by olefination reaction.[25,26]<br>2. Other electron-rich or –neutral substrates show low reactivity.[27]<br>3. Electron-deficient arenes were not reported as substrates for the electrophilic metalation mechanism.

Palladium-catalyzed non-directed C–H bond olefination enabled by ligand

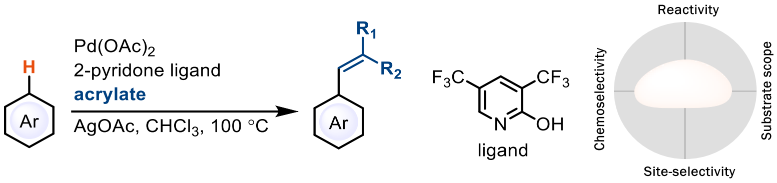

Selected examples:

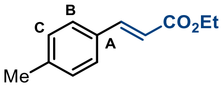

83%, A:B:C = 13.8:12.2:1

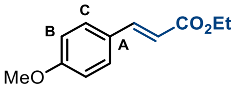

67%, A:B:C = 3.7:1.8:1

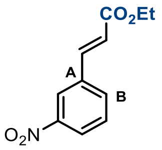

45%, A:B = 14.1:1

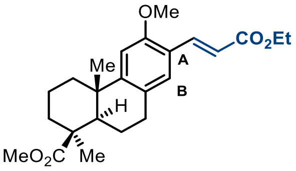

58%, A:B = 11.4:1

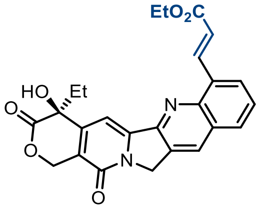

46%

Evaluation criteria	Score	Analysis[29-31]
**Reactivity**	*Moderate*	1. Most reactions are complete within 24 hours at 100 °C.[29]<br>2. The limiting reagent is arene in the ligand-promoted olefination.[29-31]<br>3. The olefination reaction requires 10 mol% $Pd(OAc)_2$ as well as 30 mol% 2-pyridone ligand.[29]
**Chemoselectivity**	*High*	1. Substrates with alcohols or carboxylic acids can be functionalized without protecting groups.[29]<br>2. Complex molecules containing sulfonamides, amides, esters, halogens, trifluoromethyls, ketones, and hetarenes can be functionalized.[29]
**Site-selectivity**	*Low*	1. The site-selectivity for ethylbenzene is *m:p* = 1.0:1.6.[29]<br>2. The site-selectivity for anisole is *p:o:m* = 3.7:1.8:1.[29]<br>3. The site-selectivity for nitrobenzene is *m:p* = 14.1:1.[29]<br>4. Mono-olefinated products are the major products, and di-olefinated products are formed as minor products when using highly reactive susbtrates.[29]
**Substrate scope**	*High*	1. Electron-rich and –poor arenes such as anisole, ethylbenzene, methyl benzoate, and nitrobenzene are suitable susbtrates.[29]<br>2. Hetarenes such as pyroles, carbazoles, (benzo)furans, (benzo)thiophenes, and quinolines can be functionalized.[29]

Palladium-catalyzed non-directed C–H bond cyanation enabled by ligand

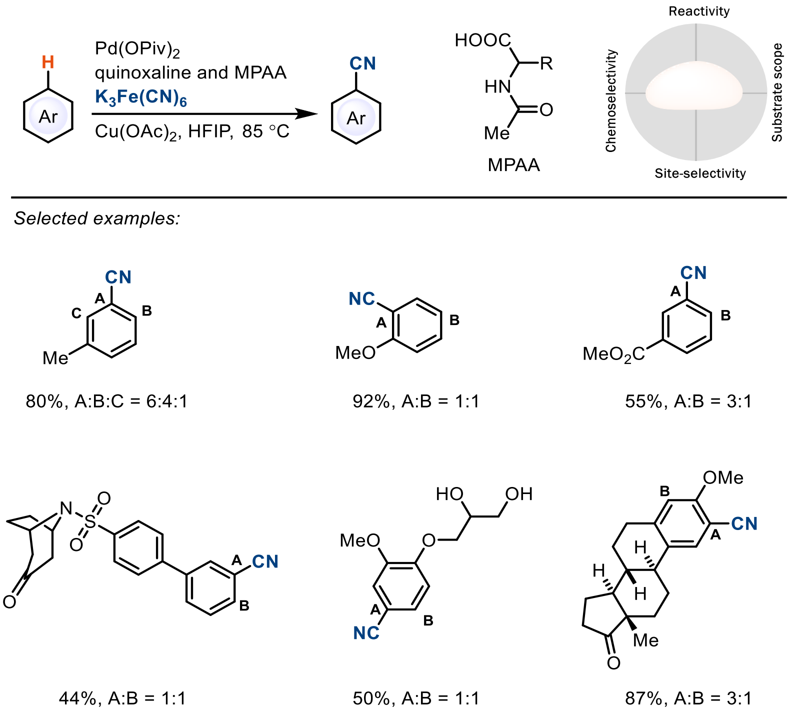

Evaluation criteria	Score	Analysis[30,32]
**Reactivity**	*Moderate*	1. Most reactions are complete within 24 hours at 85 °C.[32]<br>2. The limiting reagent is arene in the ligand-promoted cyanation.[32]<br>3. The reaction requires 10 mol% $Pd(OPiv)_2$ catalyst, and two kinds of ligands including 20 mol% Ac-Ala-OH[30] and 30 mol% quinoxaline.[32]
**Chemoselectivity**	*High*	1. Substrates with alcohols, aldehydes or indoles can be functionalized without protecting groups.[32]<br>2. Complex molecules containing sulfonamides, amides, esters, ketones, aldehydes, halogens, trifluoromethyls, and hetarenes can be functionalized.[32]
**Site-selectivity**	*Low*	1. The site-selectivity for toluene is *m*:*p*:*o* = 6:4:1.[32]<br>2. The site-selectivity for anisole is *o*:*p* = 1:1.[32]<br>3. The site-selectivity for methyl benzoate is *m*:*p* = 3:1.[32]
**Substrate scope**	*High*	1. Electron-rich and –poor arenes such as anisole, ethylbenzene, chlorobenzene, and methyl benzoate are successfully cyanated.[32]<br>2. Electron-rich hetarenes such as pyroles, indoles, thiophenes, and 2,5-dimethoxypyridine can be functionalized.[32]

Palladium-catalyzed *ortho* C–H bond hydroxylation of aryl carboxylic acids

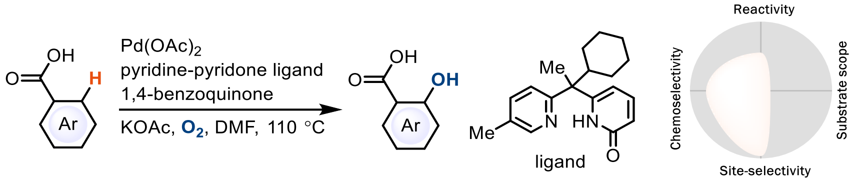

Selected examples:

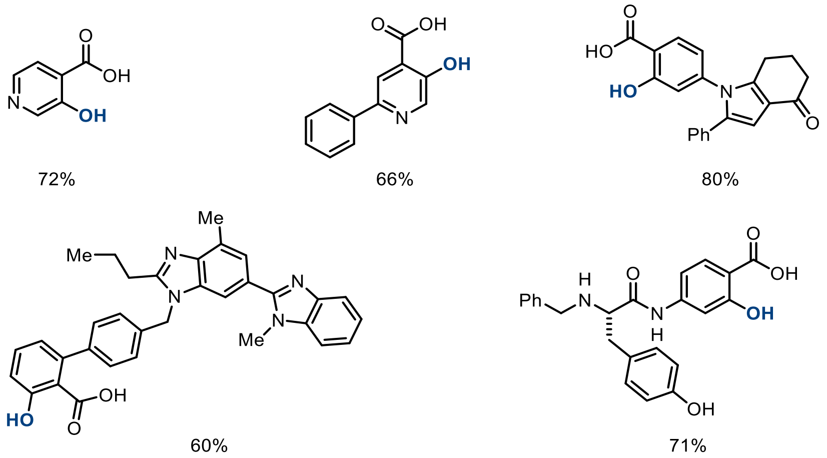

Evaluation criteria	Score	Analysis[33,34]
**Reactivity**	*Moderate*	1. Most reactions are complete within 24 hours at 110 °C.[33]<br>2. The reaction requires 1 atm of O_2.[33] Conventional oxygenation without the pyridine-pyridone ligand requires high pressure of O_2 (5 atm) to ensure sufficient reactivity.[34]<br>3. The oxygenation reaction requires 10 mol% $Pd(OAc)_2$ catalyst and 10 mol% pyridine-pyridone ligand.[33]
**Chemoselectivity**	*High*	1. Substrates with phenols, amines, or carboxylic acids can be functionalized without protecting groups.[33]<br>2. Complex molecules containing halogens, amides, ketones, amines, phenols, sulfonamides, carboxylic acids and hetarenes can be functionalized.[33]
**Site-selectivity**	*Excellent*	1. High site-selectivity is achieved in the presence of a range of commonly used directing groups including –NHAc, –NHBoc, aldehydes, and hetarenes.[33]<br>2. In the presence of one of the strongest directing groups for C–H activation, 2-pyridyl group, the carboxyl group completely overcomes the 2-pyridyl-directing effect in the oxygenation reaction.[33]<br>3. High site-selectivity is observed in almost all cases.
**Substrate scope**	*Low*	1. Aryl carboxylic acid derivatives with electron-rich or –poor arenes can be functionalized.[33]<br>2. Hetaryl carboxylic acids with benzothiophene, benzothiazole, benzofuran, benzodioxane, morpholine, pyrrole, or carbazole rings are successfully hydroxylated.[33]<br>3. Arene substrates without carboxylic acid directing groups were not reported as substrates.

Palladium-catalyzed remote C–H bond olefination of quinoline via template

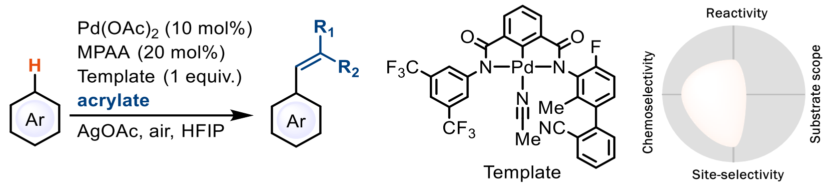

Selected examples:

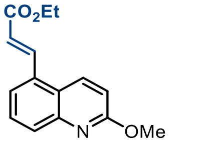
67%, C5:others = 84:16

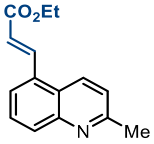
69%, C5:others = 85:15

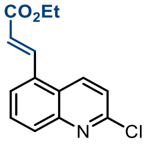
57%, C5:others = 88:12

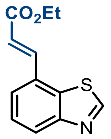
with another template
51%, C7:others = 88:12

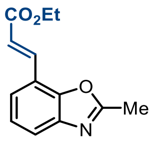
with another template
81%, C7:others = 87:13

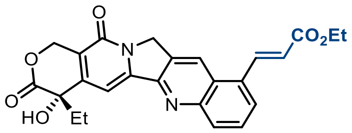
49%, C5:others = 89:11

Evaluation criteria	Score	Analysis[35,36]
**Reactivity**	*Moderate*	1. Most reactions are complete within 48 hours at 80 °C.[35]<br>2. The remote olefination reaction requires 10 mol% $Pd(OAc)_2$ and 20 mol% Ac-Gly-OH ligand.[35]<br>3. For quinoline substrates, 1.0 equivalent of template is required to ensure the remote functionalization reactivity.[35]
**Chemoselectivity**	*High*	1. Quinolines featuring esters, halogens, trifluoromethyls, amides, or hetarenes can be functionalized via the template strategy.[35]<br>2. Substrates with alcohol groups can be functionalized without protecting groups.[35]<br>3. The remote C–H bond olefination can be applied to several pharmaceuticals.[35]
**Site-selectivity**	*High*	1. The site-selectivity for quinoline is C5:others = 90:10.[35]<br>2. The site-selectivity for benzothiazole is C7:others = 88:12.[35]<br>3. The site-selectivity for 3-phenylpyridine is *m*:(*o*+*p*) = 95:5 on the phenyl ring.[35]
**Substrate scope**	*Low*	1. Quinoline, quinoxaline, benzoxazole, and benzothiazole derivatives can be functionalized with different templates.[35]<br>2. The template strategy is not feasible in the absence of an appropriate coordinating functional group.[36]

Palladium-catalyzed remote C–H bond arylation of quinoline via template and norbornene

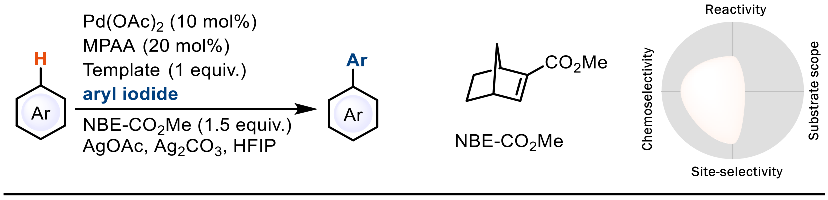

Selected examples:

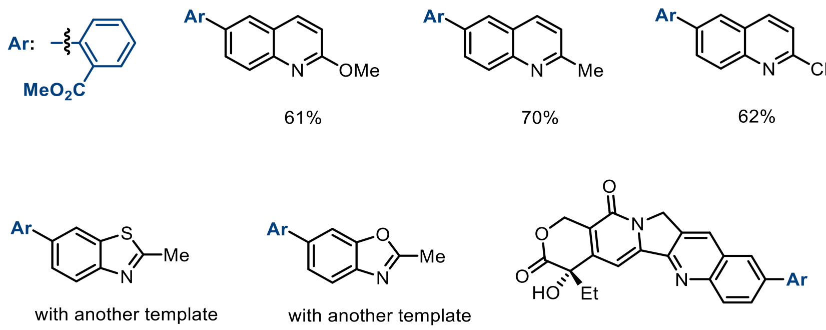

Evaluation criteria	Score	Analysis[36,37]
**Reactivity**	*Moderate*	1. Most reactions are complete within 24 hours at 80 °C.[37]<br>2. The remote arylation reaction requires 10 mol% $Pd(OAc)_2$ as well as 20 mol% Ac-Gly-OH ligand.[37]<br>3. For quinoline substrates, 1.0 equivalents of template is required to ensure the remote functionalization reactivity.[37]
**Chemoselectivity**	*High*	1. Quinolines featuring esters, halogens, trifluoromethyls, amides, or hetarenes can be functionalized by cooperation of templates and norbornene.[37]<br>2. Substrates with alcohols can be functionalized without protecting groups.[37]<br>3. The remote C–H bond arylation can be applied to the functionalization of several pharmaceuticals.[37]
**Site-selectivity**	*High*	1. In the reaction of quinoline, no undesired consitutional isomer was isolated.[37]<br>2. In the reaction of 3-methyl-isoquinoline, 61% of C5 functionalization product as well as less than 5% over-arylated product were observed.[37]
**Substrate scope**	*Low*	1. Quinoline, isoquinoline, benzoxazole, benzothiophene, indazole, or quinoxaline derivatives can be functionalized with different templates.[37]<br>2. The template and norbornene cooperation strategy is not feasible in the absence of an appropriate coordinating functional group.[36]

Ruthenium-catalyzed alkylation of C–H bonds

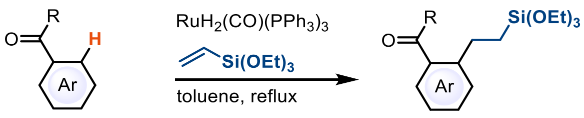

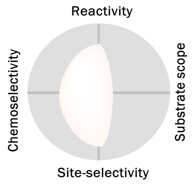

Selected examples:

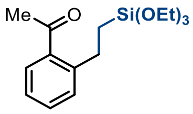

75%

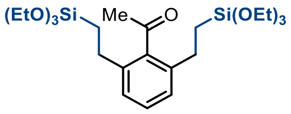

with 3 equiv. alkene
94%

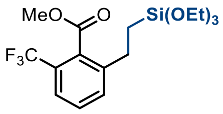

97%

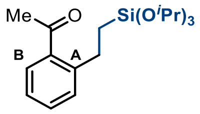

from

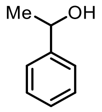

with $RuCl_3$•xH_2O, P(4-$CF_3C_6H_4$)$_3$
96%, mono:di = 63:37

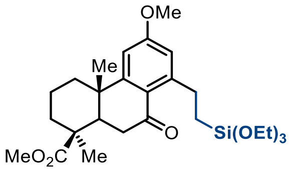

90%

Evaluation criteria	Score	Analysis[38-41]
**Reactivity**	*High*	1. The alkylation was performed in refluxing toluene (b.p. = 110 °C) in the seminal example,[38] but the reaction was complete in 48 h at 23 °C when a chelated ruthenium catalyst was applied.[39]<br>2. The reaction requires 2–6 mol% ruthenium catalyst.[38,39]<br>3. Arenes are limiting reagents in the alkylation reaction, and the reaction requires 1–5 equivalents of alkenes.[38]
**Chemoselectivity**	*Moderate*	1. Aryl ketone substrates with esters, tertiary amines, sulfonamides, silyl groups, or hetarenes can undergo the alkylation reactions.[38]<br>2. When 1-phenylethanol was applied as substrate, alcohol dehydrogenation is observed (alkylated ketone, 96% yield).[39]<br>3. The ketone-directed C−H alkylation method can be employed to derivatize diterpene natural products.[40]
**Site-selectivity**	*High*	1. When acetophenone and 2 equivalents of triethoxyl(vinyl)silane was applied as the substrates, the mono:di functionalization selectivity changes from 82:18 to 98:2 when different ruthenium catalysts were used.[38,39]<br>2. When excess alkene was applied, di-alkylation species are the major product.[39]
**Substrate scope**	*Low*	1. Aryl ketone derivatives with both electron-rich and –poor arenes can be functionalized.[38,39]<br>2. Aryl esters and aldehydes can be successfully alkylated.[39]<br>3. Ruthenium-catalyzed late-stage alkylation of arenes without directing groups were not reported.[41]

Iridium-catalyzed borylation of C–H bonds

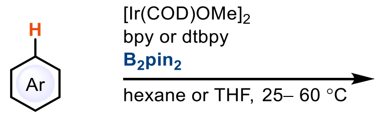

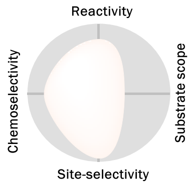

Selected examples:

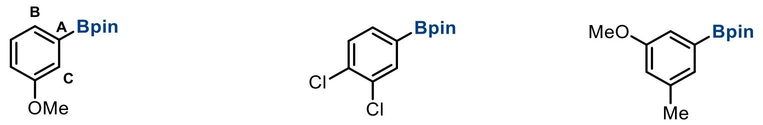

95%, A:B:C = 74:25:1 83% 72%

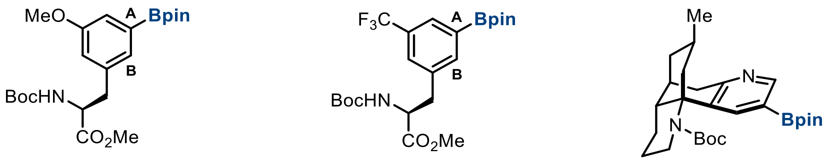

84%, A:others > 98:2 84%, A:others > 98:2 75%

Evaluation criteria	Score	Analysis[42-44]
**Reactivity**	*High*	1. The original borylation reaction requires high temperature (80 °C) and solvent amount of arene substrates (60 equiv.) in early examples.[42] Modern versions use arene as the limiting reagent, and the reactions are complete in 24 h at 25 °C .[43]<br>2. Most reactions require 3 mol% iridium catalyst, but the metal catalyst can reach >300 turnovers for the functionalization of benzene.[43]
**Chemoselectivity**	*High*	1. The borylation can be applied to complex molecules containing halogens, tertiary amines, trifluoromethyls, esters, nitriles, and hetarenes.[42-44]<br>2. Substrates with indoles, pyroles or ketones can be functionalized without protecting groups.[42-44]<br>3. The iridium catalyzed C–H functionalization has been applied to the total synthesis of several complex molecules such as (+)-Complanadine A.[44]
**Site-selectivity**	*Excellent /Low*	1. For 1,3-disubstituted arenes and several 1,2-disubstituted arenes, the site-selectivity is excellent. For example, constitutional isomers were not observed when 1,3-dichlorobenzene was applied.[43]<br>2. For mono substituted arenes, the site-selectivity is low. For examples, the site-selectivity for anisole is *m:p:o* = 74:25:1.[42]
**Substrate scope**	*Low /High*	1. The substrate scope of the site-selective borylation low, including symmetrically substituted 1,2- and 1,4-substituted arenes, 1,3-substituted arenes, benzothiophenes, benzofurans, and indoles.[43]<br>2. The substrate scope of unselective borylation is high, including but not limited to mono-substituted arenes with electron-donating or –withdrawing groups.[42,43]

Iron-catalyzed hydrogen isotope exchange (HIE) reaction

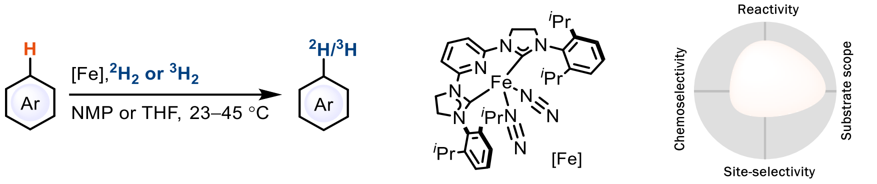

Selected examples:

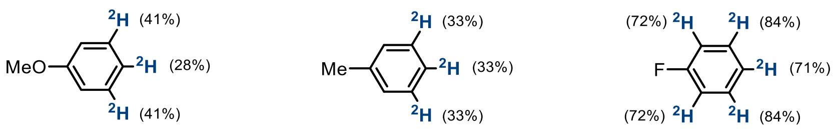

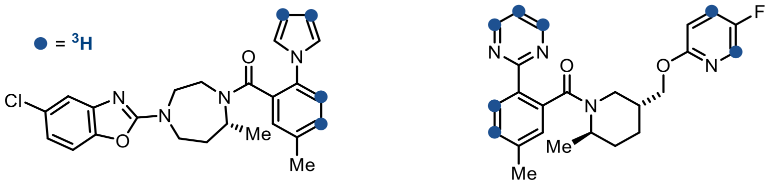

15.2 Ci mmol 57 Ci mmol

Evaluation criteria	Score	Analysis[45-47]
**Reactivity**	*High*	1. Most reactions are complete within 24 h at 23–45 °C.[45]<br>2. The deuteration reaction is carried out with 1 atm of deuterium gas. The tritiation reaction can take place with low-pressure (120 mmHg) of tritium gas.[45]<br>3. The loading of the iron catalyst is 1 mol%.[45]
**Chemoselectivity**	*Moderate*	1. The HIE reaction can be applied to complex molecules containing secondary and tertiary amines, halogens, sulfonamides, amides, trifluoromethyls, esters, nitriles, and hetarenes.[45]<br>2. Substrates with carboxylic acids or amines can be functionalized without protecting groups.[45]<br>3. Substrates with ketones, aldehyde or alcohols are not reported.[45-47]
**Site-selectivity**	*Low*	1. The ratio of deuterium incorporation for toluene is 33% at the *meta* position and 33% at the *para* position.[45]<br>2. The ratio of deuterium incorporation for anisole is 41% at the *meta* position and 28% at the *para* position.[45]<br>3. The ratio of deuterium incorporation for trifluorobenzene is 90% at the *meta* position and 90% at the *para* position.[45]
**Substrate scope**	*Excellent*	1. Electron-rich and –poor arenes such as anisole, toluene, and trifluorobenzene are all suitable substrates.[45]<br>2. Electron-rich and –poor hetarenes such as pyrole, imidazole, pyrazole, triazole, indole, thiophene, quinoline, and pyridine can be functionalized.[45]

Oxygenation of arenes with phthaloyl peroxide

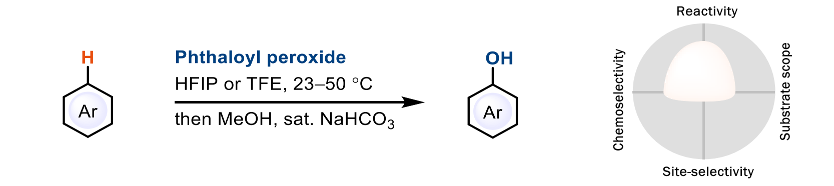

Selected examples:

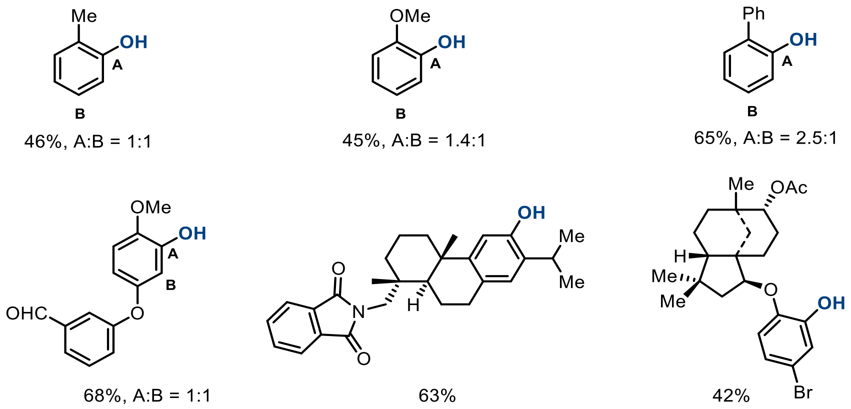

Evaluation criteria	Score	Analysis[48]
**Reactivity**	*High*	1. Most electron-rich arene substrates can be oxygenated at 23 °C within 3–24 h.[48]<br>2. Elevated temperature (40–50 °C) is required when electron-neutral arene substrates are employed.[48]<br>3. For most substrates, 1.3 equivalents of phthaloyl peroxide is required to achieve high conversion.[48]
**Chemoselectivity**	*Moderate*	1. Oxygenation with peroxides usually suffer from overoxidation or benzylic oxidation reactions,[11] which were not observed in the oxygenation reaction via phthaloyl peroxide.[48]<br>2. Complex molecules containing esters, sulfonates, halogens, trifluoromethyls, nitriles, aldehydes, epoxides, and hetarenes can be functionalized.[48]<br>3. Substrates with unprotected amines, alcohols or carboxylic acids were not reported.
**Site-selectivity**	*Low*	1. The site-selectivity for toluene is *o*:*p* = 1:1.[48]<br>2. The site-selectivity for *n*-butylbenzene is *o*:*p* = 1:1.[48]<br>3. The site-selectivity for anisole is *o*:*p* = 1.4:1.[48]
**Substrate scope**	*Moderate*	1. Both electron-rich and –neutral arenes can be functionalized.[48]<br>2. Electron-deficient arenes such as chlorobenzene or methylbenzoate are not reported as substrates for the oxygenation reaction.[48]

Palladium-catalyzed TEDAylation of arenes

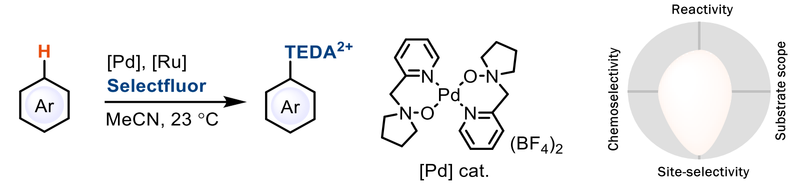

Selected examples:

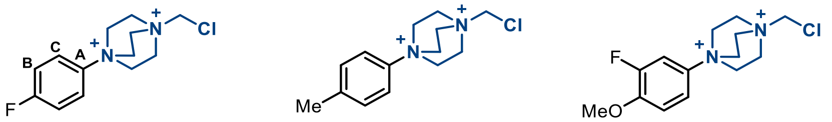

88%, A:others > 99:1 85% 86%

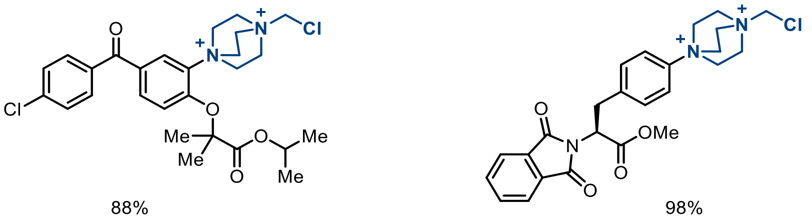

88% 98%

Evaluation criteria	Score	Analysis[49,50]
**Reactivity**	*Moderate*	1. The TEDAylation reaction requires 2.5 mol% palladium catalyst and 7.5 mol% $[Ru(bpy)_3](PF_6)_2$.[49]<br>2. Most reactions are complete within 24 h at 23 °C for electron-rich arene substrates.[49]<br>3. Elevated temperature (40–45 °C) is necessary for electron-poor arenes such as chlorobenzene.[49]
**Chemoselectivity**	*Moderate*	1. It was reported that $TEDA^{\bullet+}$ can cleave sp^3 C–H bonds, however, no such side reactions were reported in arene TEDAylation reaction.[49]<br>2. The TEDAylation reaction can be applied to molecules containing amides, esters, ketones, trifluoromethyls, sulfonamides, halogens, and hetarenes.[49,50]<br>3. Substrates with unprotected amines, alcohols or carboxylic acids were not reported.
**Site-selectivity**	*Excellent*	1. The site-selectivity for fluorobenzene is *p*:*o* > 99:1.[49]<br>2. Constitutional isomers were not observed in the TEDAylation reaction of toluene.[49]<br>3. When the *para*-position of arenes is substituted, the reaction is *ortho*-selective.[49,50]
**Substrate scope**	*Moderate*	1. Both electron-rich and –neutral arenes can be functionalized.[49]<br>2. Electron-deficient arenes such as methylbenzoate were not reported as substrates for the TEDAylation reaction.[49]<br>3. Hetarenes such as thiophenes, quinolines, and 2-methoxypyridine are successfully functionalized.[49,50]

Electrochemical trifluoromethylation of hetarene with zinc sulfinate reagents

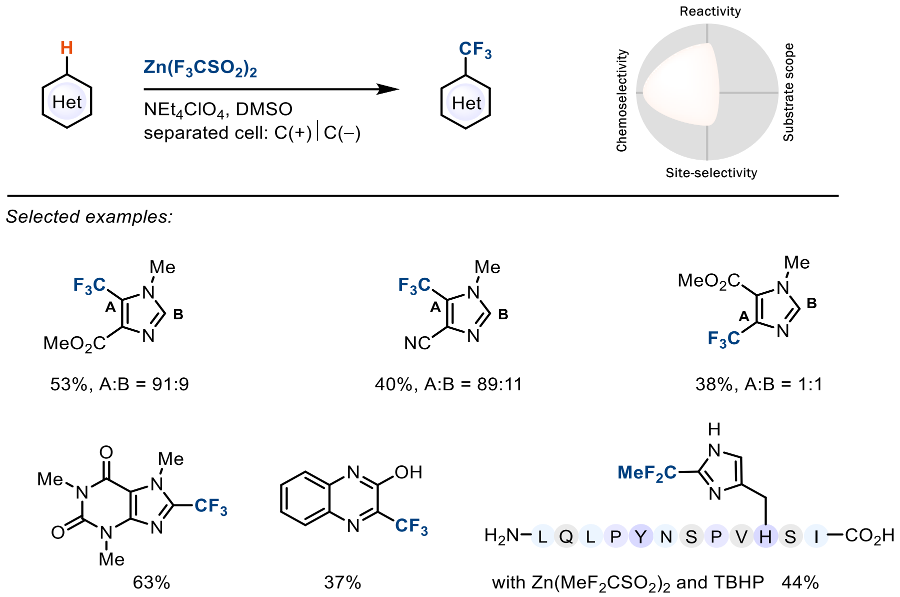

Evaluation criteria	Score	Analysis[51-53]
**Reactivity**	*High*	1. Most of the reactions are complete in 8 h at 25 °C.[51]<br>2. A constant current of 25 mA is applied, and the cell potential drifts from +0.7 V to +1.3 V during the reaction.[51]
**Chemoselectivity**	*Excellent*	1. Electrochemical initiation avoids the use of peroxides.[52,53]<br>2. Molecules containing esters, nitriles, nitros, halogens, alcohols, trifluoromethyls, ketones, amines, and hetarenes can be trifluoromethylated.[51-53]<br>3. Substrates with alcohols, phenols or amines can be functionalized without protecting groups.[51]<br>4. The functionalization reaction can tolerate all 20 natural amino acid residues, except free cysteine.[53]
**Site-selectivity**	*Moderate*	1. The site-selectivity for 1-methyl-4-cyano-imidazole is C5:C2 = 89:11.[51]<br>2. Constitutional isomers were not observed in the trifluoromethylation reaction of 1-methyl-pyrazole.[51]<br>3. For most substituted pyrole derivates, only C2 functionalization products were observed.[51]
**Substrate scope**	*Low*	1. Electron-rich hetarenes such as pyrole, pyrazole, imidazole, indole or benzothiazole derivatives can be trifluoromethylated by the approach.[51]<br>2. Electron-poor hetarenes such as diazine, quinoxaline or pyridine derivatives can be functionalized.[51-53]<br>3. Functionalized arenes were not reported as substrates for the reaction.

Amination of tyrosine via photoredox catalysis

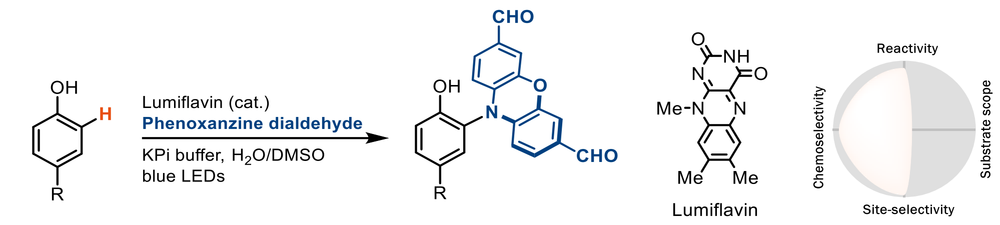

Selected examples:

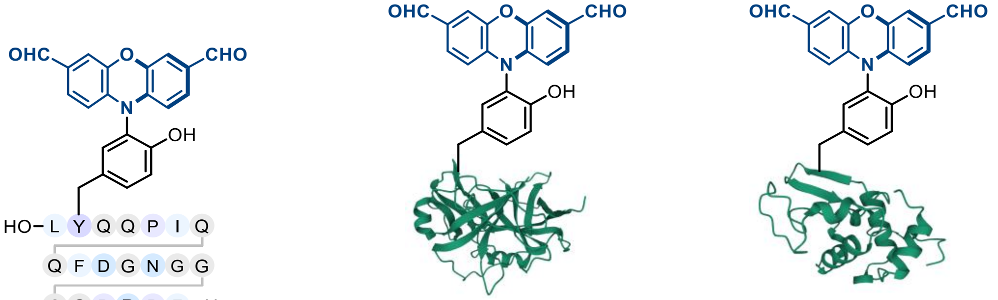

Bivalirudin (2.2 kDa)
95% conversion

Chymotrypsinogen A (25.7 kDa)
4 tyrosines
76%, Y146/Y171 = 10:1

Human lysozyme (14.4 kDa)
6 tyrosines
76%, Y45 single product

**Evaluation criteria**	**Score**	**Analysis**[54,55]
**Reactivity**	*Excellent*	1. Most reactions are complete within 0.5–5 h when irradiated with a 440 nm photoreactor at 4 °C.[54]<br>2. The reaction was carried out with 0.1 mM protein, 0.3 mM lumiflavin, and 10 mM phenoxazine dialdehyde.[54]
**Chemoselectivity**	*Excellent*	1. The amination reaction can tolerate all 20 natural amino acid residues.[54]<br>2. Proteins contain many phenols, alcohols, amines, indoles, and carboxylic acids, but the reaction can take place without protection.[54]
**Site-selectivity**	*Excellent*	1. The bioconjugation reaction is *ortho*-selective with respect to the phenol rings in tyrosine.[54]<br>2. The bioconjugation reaction is tyrosine specific among different amino acid residues.[54]<br>3. The reaction is also selective between different tyrosine residues on a protein.[54]
**Substrate scope**	*Low*	1. Polypeptides and proteins with tyrosine residues can be functionalized.[54]<br>2. The photo-induced amination reaction on other residues was not reported.[55]

Borylation of arenes with amine-borane

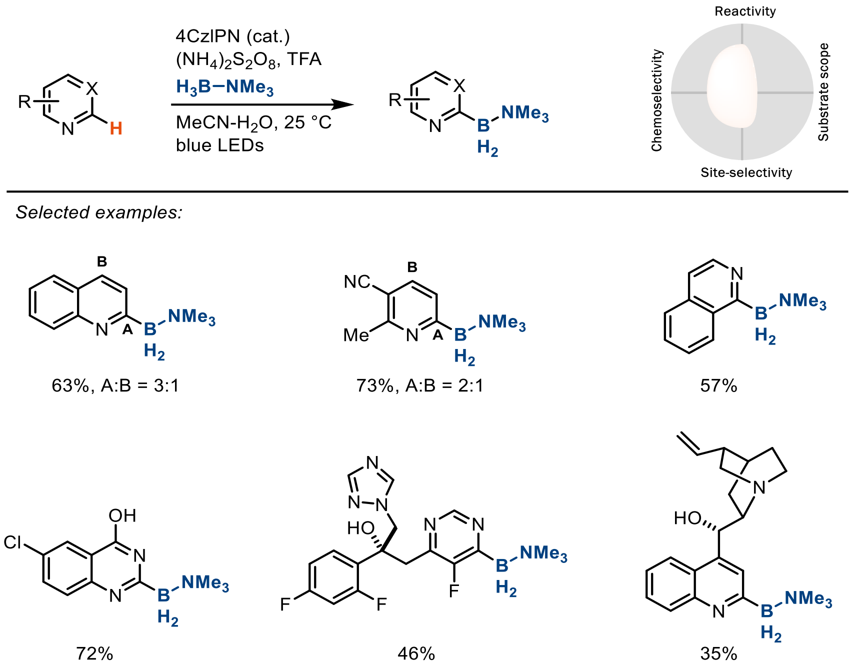

Evaluation criteria	Score	Analysis[56,57]
**Reactivity**	*High*	1. The borylation reaction is complete within 12 h at 25 °C.[56]<br>2. The borylation reaction requires 2 mol% photocatalyst and 4.0 equivalents of amino-borane to ensure high conversion.[56]<br>3. The quantum yield of the radical borylation reaction is $\Phi = 0.41$.[56]
**Chemoselectivity**	*Moderate*	1. The borylation requires persulfate as the oxidant, which may cause benzylic oxidation reactions. However, no such side reactions were reported in the radical borylation.[56]<br>2. The borylation reaction can be applied to complex molecules containing amines, sulfonamides, halogens or hetarenes.[56]<br>3. Substrates with phenols, alcohols or amines can be functionalized without protecting groups.[56]<br>4. Substrates with ketones or aldehydes were not reported.[57]
**Site-selectivity**	*Moderate*	1. The site-selectivity for quinoline is C2:C4 = 3:1.[56]<br>2. For isoquinoline, the borylation reaction is C1-selective.[56]<br>3. Only C2-substituted products can be observed when the C4 position of pyridines or quinolines are substituted.[56]
**Substrate scope**	*Low*	1. Pyridine, diazine, quinoline, isoquinoline, quinazoline, and benzothiazole derivatives can be functionalized.[56]<br>2. Functionalized arenes were not reported as substrates for the transformation.

Radiofluorination reaction of aromatic C–H bonds via photoredox catalysis

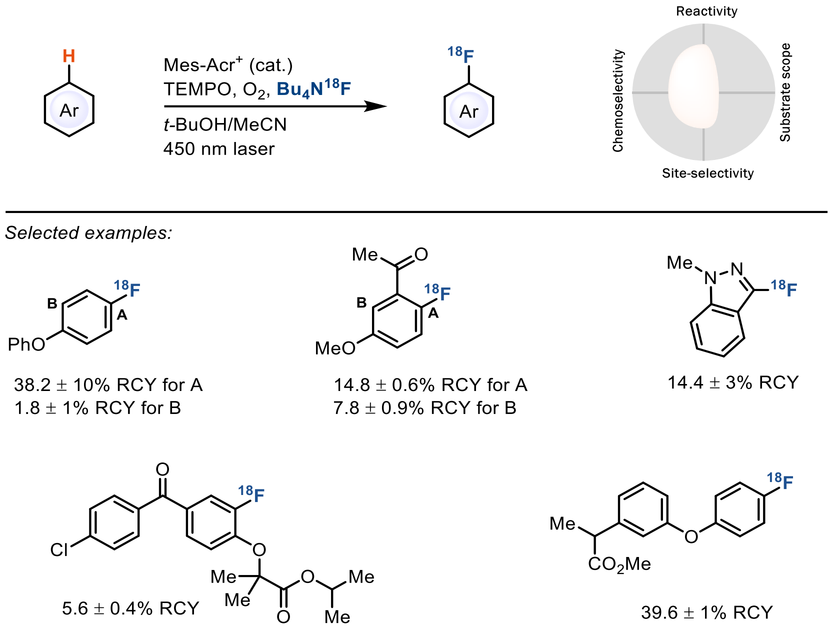

Evaluation criteria	Score	Analysis[58,59]
**Reactivity**	*High*	1. Most reactions are complete within 30 min at 0 °C when irradiated with a 450 nm laser.[58]<br>2. The reaction requires 1 atm of O_2.[58]<br>3. The loading of photocatalyst Mes-$Acr^+ClO_4^-$ is 1–5 mol%.[58]
**Chemoselectivity**	*Moderate*	1. Complex molecules containing amides, sulfonates, halogens, aldehydes, ketones, esters, and hetarenes can be functionalized.[58,59]<br>2. Substrates with unprotected amines, alcohols, carboxylic acids were not reported.[58]
**Site-selectivity**	*Moderate*	1. The site-selectivity for diphenyl ether is *p*:*o* = 17.9:1 on the phenyl ring.[58]<br>2. The site-selectivity for 3-methoxyacetophenone is C5:C2 = 1.9:1.[58]<br>3. Only *ortho*-substituted product was observed when the *para* position of an electron-rich arene is substituted.[58]
**Substrate scope**	*Low*	1. Electron-rich arenes such as anisole, indazole, benzimidazole, and benzoxazole derivatives are successfully radiofluorinated.[58]<br>2. Biphenyl or naphthalene derivatives can be functionalized.[58]<br>3. Electron-neutral or –poor arenes are not reported as substrates for the radiofluorination reaction.

Hydroxylation of aromatic C–H bonds via electrophotoredox catalysis

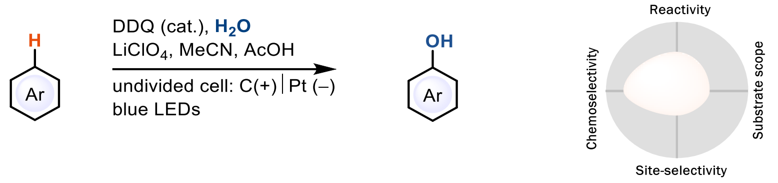

Selected examples:

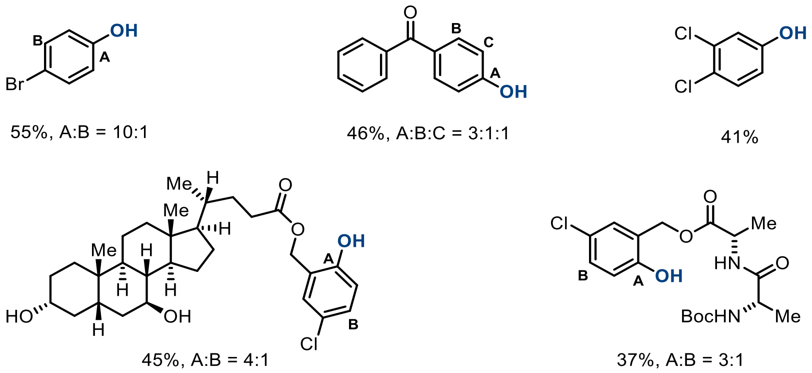

Evaluation criteria	Score	Analysis[60,61]
**Reactivity**	*Moderate*	1. The oxygenation reaction requires 10 mol% electrophotocatalyst.[60]<br>2. Most reactions are complete within 12 h under a controlled potential of 1.5 V as well as irradiation from a blue LED at 25 °C.[60]
**Chemoselectivity**	*High*	1. Oxygenation of arenes usually suffer from overoxidation or benzylic oxidation reactions,[11] which were not observed in the reactions via electrophotoredox catalysis.[60]<br>2. Complex molecules containing amides, sulfonamides, halogens, ketones, esters, and alcohols can be oxygenated.[60]<br>3. Substrates with alcohols can be functionalized without protecting groups.[60]
**Site-selectivity**	*Moderate*	1. The site-selectivity for bromobenzene is *p:o* = 10:1.[60,61]<br>2. The site-selectivity for benzophenone is *p:o:m* = 3:1:1.[60]<br>3. Constitutional isomers are not observed in the oxygenation reaction of 1,2-dichlorobenzene.[1]
**Substrate scope**	*Moderate*	1. Both electron-rich and –neutral arenes can be functionalized.[60]<br>2. Electron-poor arenes such as bromobenzene can be functionalized, but more electron-poor substrates such as methylbenzoate were not reported as substrates for the oxygenation reaction.[60]

REFERENCES

1. Hajipour, A. R.; Ruoho, A. E. Nitric acid in the presence of P_2O_5 supported on silica gel—a useful reagent for nitration of aromatic compounds under solvent-free conditions. *Tetrahedron Lett.* **2005**, *46*, 8307–8310.
2. Calvo, R.; Zhang, K.; Passera, A.; Katayev, D. Facile access to nitroarenes and nitroheteroarenes using *N*-nitrosaccharin. *Nat. Commun.* **2019**, *10*, 3410.
3. Zohuri, G. H.; Seyedi, S. M.; Sandaroos, R.; Damavandi, S.; Mohammadi, A. Novel late transition metal catalysts based on iron: synthesis, structures and ethylene polymerization. *Catal. Lett.* **2010**, *140*, 160–166.
4. Nishii, Y.; Ikeda, M.; Hayashi, Y.; Kawauchi, S.; Miura, M. Triptycenyl sulfide: A practical and active catalyst for electrophilic aromatic halogenation using N-halosuccinimides. *J. Am. Chem. Soc.* **2020**, *142*, 1621–1629.
5. Ueno, K.; Nishii, Y.; Miura, M. Electrophilic Substitution of Asymmetrically Distorted Benzenes within Triptycene Derivatives. *Org. Lett.* **2021**, *23*, 3552–3556.
6. Tanemura, K.; Suzuki, T.; Nishida, Y.; Satsumabayashi, K.; Horaguchi, T. Halogenation of Aromatic Compounds by *N*-chloro-, *N*-bromo-, and *N*-iodosuccinimide. *Chem. Lett.* **2003**, *32*, 932-933.
7. Yamamoto, K.; Li, J.; Garber, J. A.; Rolfes, J. D.; Boursalian, G. B.; Borghs, J. C.; Genicot, C.; Jacq, J.; van Gastel, M.; Neese, F.; Ritter, T. Palladium-catalysed electrophilic aromatic C–H fluorination. *Nature*, **2018**, *554*, 511–514.
8. Taylor, S. D.; Kotoris, C. C.; Hum, G. Recent advances in electrophilic fluorination. *Tetrahedron*, **1999**, *55*, 12431-12477.
9. Lal, G. S.; Pez, G. P.; Syvret, R. G. Electrophilic NF fluorinating agents. *Chem. Rev.* **1996**, *96*, 1737-1756.
10. Börgel, J.; Tanwar, L.; Berger, F.; Ritter, T. Late-stage aromatic C–H oxygenation. *J. Am. Chem. Soc.* **2018**, *140*, 16026–16031.
11. Udenfriend, S.; Clark, C. T.; Axelrod, J.; Brodie, B. B. Ascorbic Acid In Aromatic Hydroxylation: I. A Model System for Aromatic Hydroxylation. *J. Biol. Chem.* **1954**, *208*, 731−740.
12. Stang, P. J.; Anderson, A. G. Hammett and Taft substituent constants for the mesylate, tosylate, and triflate groups. *J. Org. Chem.* **1976**, *41*, 781-785.
13. Berger, F.; Plutschack, M. B.; Riegger, J.; Yu, W.; Speicher, S.; Ho, M.; Frank, N.; Ritter, T. Site-selective and versatile aromatic C–H functionalization by thianthrenation. *Nature* **2019**, *567*, 223–228.
14. Juliá, F.; Shao, Q.; Duan, M.; Plutschack, M. B.; Berger, F.; Mateos, J.; Lu, C.; Xue, X.-S.; Houk, K. N.; Ritter, T. High Site-Selectivity in Electrophilic Aromatic Substitutions: Mechanism of C–H Thianthrenation. *J. Am. Chem. Soc.* **2021**, 143, 16041–16054.
15. Shine, H. J.; Silber, J. J. Ion radicals. XXII. Reaction of thianthrenium perchlorate (C12H8S2$^{\cdot+}$ClO4^{-}) with aromatics. *J. Org. Chem.* **1971**, *36*, 2923−2926.
16. Craven, E. J.; Latham, J.; Shepherd, S. A.; Khan, I.; Diaz-Rodriguez, A.; Greaney, M. F.; Micklefield, J. Programmable late-stage C−H bond functionalization enabled by integration of enzymes with chemocatalysis. *Nat. Catal.* **2021**, *4*, 385–394.
17. Shepherd, S. A.; Menon, B. R.; Fisk, H.; Struck, A. W.; Levy, C.; Leys, D.; Micklefield, J. A structure-guided switch in the regioselectivity of a tryptophan halogenase. *ChemBioChem* **2016**, *17*, 821–824.
18. Latham, J.; Brandenburger, E.; Shepherd, S. A.; Menon, B. R.; Micklefield, J. Development of halogenase enzymes for use in synthesis. *Chem. Rev.* **2018**, *118*, 232–269.

19. Makosza, M.; Winiarski, J. Vicarious nucleophilic substitution of hydrogen. *Acc. Chem. Res.* **1987**, *20*, 282–289.

20. Zlotin, S. G.; Kislitsin, P. G.; Kucherov, F. A.; Serebryakov, E. A.; Strelenko, Y. A.; Gakh, A. A. Synthetic utilization of polynitro aromatic compounds. 5. Multi-centered reactivity pattern in reactions of 4,6-dinitro-1,2-benzisothiazoles and -isothiazol-3(2H)-ones with C-, N-, O-, S-, and F-nucleophiles. *Heterocycles* **2006**, *68*, 2483–2498.

21. Mαjkosza, M.; Danikiewicz, W.; Wojciechowski, K. Vicarious nucleophilic substitution with sulfur containing carbanions. *Phosphorus Sulfur Silicon Relat. Elem.* **1990**, *53*, 457-475.

22. Hilton, M. C.; Dolewski, R. D.; McNally, A. Selective functionalization of pyridines via heterocyclic phosphonium salts. *J. Am. Chem. Soc.* **2016**, *138*, 13806–13809.

23. Dolewski, R. D.; Hilton, M. C.; McNally, A. 4-Selective Pyridine Functionalization Reactions via Heterocyclic Phosphonium Salts. *Synlett* **2018**, *29*, 08–14.

24. Dolewski, R. D.; Fricke, P. J.; McNally, A. Site-selective switching strategies to functionalize polyazines. *J. Am. Chem. Soc.* **2018**, *140*, 8020–8026.

25. Wang, Y. J.; Yuan, C. H.; Chu, D. Z.; Jiao, L. Regiocontrol in the oxidative Heck reaction of indole by ligand-enabled switch of the regioselectivity-determining step. *Chem. Sci.* **2020**, *11*, 11042–11054.

26. Moghaddam, F. M.; Pourkaveh, R.; Karimi, A. Oxidative Heck Reaction as a Tool for Para-selective Olefination of Aniline: A DFT Supported Mechanism. *J. Org. Chem.* **2017**, *82*, 10635–10640.

27. Dams, M.; De Vos, D. E.; Celen, S.; Jacobs, P. A. Toward Waste-Free Production of Heck Products with a Catalytic Palladium System under Oxygen. *Angew. Chem., Int. Ed.* **2003**, *42*, 3512–3515.

28. Yokoyama, Y.; Matsumoto, T.; Murakami, Y. Optically active total synthesis of clavicipitic acid. *J. Org. Chem.* **1995**, 60, 1486-1487.

29. Wang, P.; Verma, P.; Xia, G.; Shi, J.; Qiao, J. X.; Tao, S.; Cheng, P. T. W.; Poss. M. A.; Farmer, M. E.; Yeung, K.; Yu, J. Q. Ligand-accelerated non-directed C–H functionalization of arenes. *Nature* **2017**, *551*, 489-493.

30. Wang, D. H.; Engle, K. M.; Shi, B. F.; Yu, J. Q. Ligand-enabled reactivity and selectivity in a synthetically versatile aryl C–H olefination. *Science* **2010**, *327*, 315–319.

31. Lafrance, M.; Rowley, C. N.; Woo, T. K.; Fagnou, K. Catalytic intermolecular direct arylation of perfluorobenzenes. *J. Am. Chem. Soc.* **2006**, *128*, 8754–8756.

32. Zhao, D.; Xu, P.; Ritter, T. Palladium-catalyzed late-stage direct arene cyanation. *Chem* **2019**, *5*, 97–107.

33. Li, Z.; Wang, Z.; Chekshin, N.; Qian, S.; Qiao, J. X.; Cheng, P. T.; Yeung, K. S.; Ewing, W. R.; Yu, J. Q. A tautomeric ligand enables directed C–H hydroxylation with molecular oxygen. *Science* **2021**, *372*, 1452–1457.

34. Zhang, Y. H.; Yu, J. Q. Pd (II)-catalyzed hydroxylation of arenes with 1 atm of O2 or air. *J. Am. Chem. Soc.* **2009**, *131*, 14654–14655.

35. Zhang, Z.; Tanaka, K.; Yu, J. Q. Remote site-selective C–H activation directed by a catalytic bifunctional template. *Nature* **2017**, *543*, 538–542.

36. Meng, G.; Lam, N. Y.; Lucas, E. L.; Saint-Denis, T. G.; Verma, P.; Chekshin, N.; Yu, J. Q. Achieving site-selectivity for C–H activation processes based on distance and geometry: a carpenter's approach. *J. Am. Chem. Soc.* **2020**, *142*, 10571–10591.

37. Shi, H.; Lu, Y.; Weng, J.; Bay, K. L.; Chen, X.; Tanaka, K.; Verma, P.; Houk, K.N.; Yu, J.Q. Differentiation and functionalization of remote C–H bonds in adjacent positions. *Nat. Chem.* **2020**, *12*, 399–404.

38. Murai, S.; Kakiuchi, F.; Sekine, S.; Tanaka, Y.; Kamatani, A.; Sonoda, M.; Chatani, N. Efficient catalytic addition of aromatic carbon-hydrogen bonds to olefins. *Nature* **1993**, *366*, 529–531.

39. Dong, Z.; Ren, Z.; Thompson, S. J.; Xu, Y.; Dong, G. Transition-metal-catalyzed C–H alkylation using alkenes. *Chem. Rev.* **2017**, *117*, 9333–9403.

40. (a) Harris, P. W. R.; Woodgate, P. D. Ruthenium-Catalysed Ortho Alkylation of Hydroxyacetophenones; the Functionalisation of Ring C Aromatic Diterpenoids. *J. Organomet. Chem.* **1996**, *506*, 339−341. (b) Harris, P. W. R.; Woodgate, P. D. Ruthenium-Catalysed Ortho Alkylation of Hydroxyacetophenones; the Functionalisation of Ring C Aromatic Diterpenoids. *J. Organomet. Chem.* **1997**, *530*, 211−223. (c) Wright, J. S.; Sharninghausen, L. S.; Preshlock, S.; Brooks, A. F.; Sanford, M. S.; Scott, P. J. Sequential Ir/Cu-Mediated Method for the Meta-Selective C–H Radiofluorination of (Hetero) Arenes. *J. Am. Chem. Soc.* **2021**, *143*, 6915-6921.

41. Lail, M.; Arrowood, B. N.; Gunnoe, T. B. Addition of arenes to ethylene and propene catalyzed by ruthenium. *J. Am. Chem. Soc.* **2003**, *125*, 7506-7507.

42. Ishiyama, T.; Takagi, J.; Ishida, K.; Miyaura, N.; Anastasi, N. R.; Hartwig, J. F. Mild iridium-catalyzed borylation of arenes. High turnover numbers, room temperature reactions, and isolation of a potential intermediate. *J. Am. Chem. Soc.* **2002**, *124*, 390–391.

43. Hartwig, J. F. Borylation and silylation of C–H bonds: a platform for diverse C–H bond functionalizations. *Acc. Chem. Res.* **2012**, *45*, 864-873.

44. (a) Meyer, F. M.; Liras, S.; Guzman-Perez, A.; Perreault, C.; Bian, J.; James, K. Functionalization of Aromatic Amino Acids via Direct C−H Activation: Generation of Versatile Building Blocks for Accessing Novel Peptide Space. *Org. Lett.* **2010**, *12*, 3870–3873. (b) Fischer, D. F.; Sarpong, R. Total synthesis of (+)-complanadine A using an iridium-catalyzed pyridine C− H functionalization. *J. Am. Chem. Soc.* **2010**, *132*, 5926-5927.

45. Yu, R. P.; Hesk, D.; Rivera, N.; Pelczer, I.; Chirik, P. J. Iron-catalysed tritiation of pharmaceuticals. *Nature* **2016**, *529*, 195–199.

46. Arevalo, R.; Chirik, P. J. Enabling two-electron pathways with iron and cobalt: from ligand design to catalytic applications. *J. Am. Chem. Soc.* **2019**, *141*, 9106-9123.

47. Corpas, J.; Viereck, P.; Chirik, P. J. C (sp2)–H Activation with Pyridine Dicarbene Iron Dialkyl Complexes: Hydrogen Isotope Exchange of Arenes Using Benzene-d 6 as a Deuterium Source. *ACS Catal.* **2020**, *10*, 8640-8647.

48. Yuan, C.; Liang, Y.; Hernandez, T.; Berriochoa, A.; Houk, K. N.; Siegel, D. Metal-free oxidation of aromatic carbon–hydrogen bonds through a reverse-rebound mechanism. *Nature* **2013**, *499*, 192–196.

49. Boursalian, G. B.; Ham, W. S.; Mazzotti, A. R.; Ritter, T. Charge-transfer-directed radical substitution enables para-selective C–H functionalization. *Nat. Chem.* **2016**, *8*, 810–815.

50. Serpier, F.; Pan, F.; Ham, W. S.; Jacq, J.; Genicot, C.; Ritter, T. Selective Methylation of Arenes: A Radical C−H Functionalization/Cross-Coupling Sequence. *Angew. Chem., Int. Ed.* **2018**, *130*, 10857–10861.

51. O'Brien, A. G.; Maruyama, A.; Inokuma, Y.; Fujita, M.; Baran, P. S.; Blackmond, D. G. Radical C−H Functionalization of Heteroarenes under Electrochemical Control. *Angew. Chem., Int. Ed.* **2014**, *53*, 11868–11871.

52. (a) Ji, Y.; Brueckl, T.; Baxter, R. D.; Fujiwara, Y.; Seiple, I. B.; Su, S.; Blackmond, D. G.; Baran, P. S. Innate C–H trifluoromethylation of heterocycles. *Pro. Nat. Aca. Sci.* **2011**, *108*, 14411–14415. (b) Fujiwara, Y.; Dixon, J. A.; O'Hara, F.; Funder, E. D.; Dixon, D. D.; Rodriguez, R. A.; Baxter, R. D.; Herle, B.; Sach, N.; Collins, M. R.; Ishihara, Y.; Baran, P. S. Practical and innate carbon–hydrogen functionalization of heterocycles. *Nature* **2012**, *492*, 95–99.

53. Noisier, A. F.; Johansson, M. J.; Knerr, L.; Hayes, M. A.; Drury III, W. J.; Valeur, E.; Valeur, E.; Malins, L. R.; Gopalakrishnan, R. Late-Stage Functionalization of Histidine in Unprotected Peptides. *Angew. Chem., Int. Ed.* **2019**, *131*, 19272-19278.

54. For selected examples on tyrosine modification, see: (a) Song, C.; Liu, K.; Wang, Z.; Ding, B.; Wang, S.; Weng, Y.; Chiang, C-W; Lei, A. Electrochemical oxidation induced selective tyrosine bioconjugation for the modification of biomolecules. *Chem. Sci.* **2019**, *10*, 7982–7987. (b) Li, B. X.; Kim, D. K.; Bloom, S.; Huang, R. Y. C.; Qiao, J. X.; Ewing, W. R.; Oblinsky, D. G.; Scholes, G. D.; MacMillan, D. W. C. Site-selective tyrosine bioconjugation via photoredox catalysis for native-to-bioorthogonal protein transformation. *Nat. Chem.* **2021**, *13*, 902–908.

55. (a) Sletten, E. M.; Bertozzi, C. R. Bioorthogonal chemistry: fishing for selectivity in a sea of functionality. *Angew. Chem., Int. Ed.* **2009**, *48*, 6974-6998. (b) Tong, H. R.; Li, B.; Li, G.; He, G.; Chen, G. Postassembly modifications of peptides via metal-catalyzed C–H functionalization. *CCS Chem.* **2021**, *3*, 1797-1820.

56. Kim, J. H.; Constantin, T.; Simonetti, M.; Llaveria, J.; Sheikh, N. S.; Leonori, D. A radical approach for the selective C–H borylation of azines. *Nature* **2021**, *595*, 577–583.

57. Taniguchi, T. Boryl radical addition to multiple bonds in organic synthesis. *Eur. J. Org. Chem.* **2019**, *2019*, 6308-6319.

58. Chen, W.; Huang, Z.; Tay, N. E.; Giglio, B.; Wang, M.; Wang, H.; Wu, Z.; Nicewicz, D. A.; Li, Z. Direct arene C–H fluorination with $^{18}F^{-}$ via organic photoredox catalysis. *Science* **2019**, *364*, 1170–1174.

59. Romero, N. A.; Margrey, K. A.; Tay, N. E.; Nicewicz, D. A. Site-selective arene CH amination via photoredox catalysis. *Science* **2015**, *349*, 1326-1330.

60. Huang, H.; Lambert, T. H. Electrophotocatalytic C−H Heterofunctionalization of Arenes. *Angew. Chem., Int. Ed.* **2021**, *60*, 11163–11167.

61. Ohkubo, K., Fujimoto, A.; Fukuzumi, S. Visible-light-induced oxygenation of benzene by the triplet excited state of 2, 3-dichloro-5, 6-dicyano-p-benzoquinone. *J. Am. Chem. Soc.* **2013**, *135*, 5368–5371.
